# Strongly regulated transcription factors exert an outsized influence in microRNA-regulated networks

**DOI:** 10.1186/s12964-025-02626-w

**Published:** 2025-12-27

**Authors:** Laura Sourdin, Julie M. Bracken, Philip A. Gregory, Nora Feldker, Thomas Brabletz, Simone Brabletz, Yeesim Khew-Goodall, Gregory J. Goodall, Katherine A. Pillman, Cameron P. Bracken

**Affiliations:** 1 Centre for Cancer Biology, An Alliance of SA Pathology and University of South Australia, Bradley Building, North Terrace, Adelaide, SA 5000 Australia; 2 School of Medicine, Discipline of Medicine, University of Adelaide, Adelaide, SA Australia; 3 Department of Experimental Medicine 1, Nikolaus-Fiebiger-Center for Molecular Medicine, Friedrich-Alexander-University Erlangen-Nürnberg, Erlangen, Germany; 4 Department of Nephropathology, Institute of Pathology, Friedrich-Alexander-Universität Erlangen-Nürnberg, Erlangen, Germany; 5 School of Biological Sciences, Faculty of Sciences, University of Adelaide, Adelaide, SA Australia; 6 ACRF Cancer Genomics Facility, Centre for Cancer Biology, SA Pathology, Adelaide, Australia

**Keywords:** MicroRNA, Gene regulation, Transcription factor, MiR-200, Bi-stable switch

## Abstract

Identifying the most biologically meaningful microRNA (miRNA) targets remains challenging, as predictive and biochemical methods yield many weak or non-productive interactions. Transcription factors (TFs) are enriched among miRNA targets and amplify miRNA effects through their broad regulatory influence. Frequently, these same TFs also regulate the miRNA, forming double negative feedback loops that enforce bistable gene expression and cell-fate decisions. We investigated this regulatory motif by focusing on reciprocal repression between the miR-200 family and ZEB1/2, which governs epithelial–mesenchymal plasticity. Employing a system isolating ZEB-dependent effects of miR-200c and combining Weighted Gene Co-expression Network Analysis (WGCNA) with Exon-Intron Split Analysis (EISA), as well as functional cell biology assays, we show this circuit reinforces mutually exclusive epithelial and mesenchymal states through complex networks of intertwined direct and indirect, transcriptional and post-transcriptional, ZEB-dependent and independent mechanisms. Our findings highlight how miRNA-TF feedback loops can act as bistable switches to lock cell identity and emphasize the pivotal role of strongly regulated TFs within miRNA target networks.

**Supplementary Information:**

The online version contains supplementary material available at 10.1186/s12964-025-02626-w.

## Introduction

MicroRNAs (miRNAs) control gene expression by targeting messenger RNA (mRNA) for degradation or translational repression. In co-ordination with an argonaute (AGO) protein, the RNA-Induced Silencing Complex (RISC; comprised of AGO, an AGO-bound miRNA and accessory proteins), is guided to mRNAs via complementary base pairing between the miRNA “seed sequence” and mRNA target sites that are primarily located within 3’ untranslated regions (3’UTR). In this manner, miRNAs exert complex regulation on gene networks through the collective actions of hundreds of miRNAs, regulating to a greater or lesser extent the majority of the transcriptome [1]. Thus, miRNAs play pivotal roles in various physiological processes such as cell proliferation, differentiation, homeostasis and apoptosis and their dysregulated expression is associated with a spectrum of diseases including cancer, where they can contribute to tumour initiation, progression, and metastasis.

In the field of miRNA biology, a significant challenge lies in accurately discerning the genes for which miRNA targeting holds biological relevance. This is challenging for several reasons, including the identification of the targets themselves. Given that miRNAs only require a short (6–8 nucleotide) region of sequence complementarity, there are thousands of potential targets for each miRNA. Methodological advances in the identification of RNAs physically bound to miRNAs have reduced the reliance on theoretical target prediction [2, 3]. However, the non-quantitative nature of the detection, and the large numbers in which they are found, do little to narrow down which are the most significant. This complexity is exacerbated by questions of stoichiometry such as whether the interaction remains important if the amount of target vastly outweighs the available miRNA. Moreover, it is often challenging to detect the subtle effects of miRNAs, particularly when they act at the translational level and thus evade high-throughput transcriptomics. Adding still to the difficulty of identifying targets of biological significance are the varied manner through which miRNAs can regulate genetic networks. For example, is prominent regulation of a specific gene important, or does significance emerge from the cross-targeting of multiple genes across common pathways? Perhaps even modest gene suppression may be important if genes with co-operative roles are being simultaneously suppressed.

It is well established that miRNAs frequently target transcription factors (TFs), and conversely, these TFs often reciprocally target the same miRNAs, either positively or negatively [4–8]. The interplay between TFs and miRNAs also extends to the co-targeting of additional genes, resulting in an array of coherent and incoherent feedback and feedforward loops that serve to buffer biological noise, maintain gene expression within defined limits, or act as switches between mutually exclusive states [9, 10]. Given the far-reaching consequences of their regulation, TFs may constitute an especially critical type of miRNA target gene. Recent observations, including from our laboratory, suggest that a considerable proportion of the change in gene expression that follows miRNA perturbation occurs at the transcriptional level [11, 12]. This implies the regulation of specific TFs may be a key feature of miRNA function; though the significance of the downstream network of transcriptional targets is often overlooked on account of the tendency to focus on direct target genes.

With these considerations in mind, we set out to identify to what extent prominent TF targets can be responsible for the biological actions of a miRNA. If such consequential targets dominate the output of miRNA perturbation, then a clear argument would exist for their prioritization and by extension, bring into question the significance of numerous reports in which biological significance is tied to the modest suppression of otherwise unremarkable target genes. To investigate, we focused in depth on the reciprocal negative feedback loop operating between the epithelial enforcing miR-200 family of miRNAs and the mesenchymal promoting ZEB family of TFs that controls epithelial to mesenchymal transition (EMT) and thus, the metastatic capacity of cancer cells [13, 14]. By constructing cells in which the capacity to directly regulate ZEB was uncoupled from all other aspects of miR-200 function, we thus interrogated to what extent is the impact of miR-200 executed through, or independent of, specific targeting of ZEB? Correspondingly, to what extent do the hundreds of ZEB-independent targets contribute to the role of this miRNA, given that the reciprocal feedback loop between miR-200 and ZEB is so prominent?

A hallmark of MET is the upregulation of epithelial-specific genes (CDH1, ESRP1 etc.). We find that the capacity of miR-200c to drive epithelial gene expression is dependent upon its ability to suppress the ZEB TFs, however the requirement to suppress ZEB in other aspects of miR-200c function is variable, ranging from partial ZEB-dependency in the suppression of cell migration by miR-200c, through to ZEB-independency with regard to miR-200c inhibiting MMP-mediated ECM-degradation and promoting adhesion. To examine these effects more systemically, we also produced and interrogated transcriptomic data using both gene co-expression network analysis (WGCNA) and Exon-Intron Split Analysis (EISA), allowing us to uncover a complex continuum of gene expression whereby genes enriched for EMT-related processes are controlled and reinforced by both arms of the miRNA - TF motif via combinations of direct and indirect, transcriptional and post-transcriptional regulation.

Our work argues for the importance of focusing on strongly-regulated TF targets when assessing miRNA function, and exemplifies how mutually exclusive, bi-stable phenotypes are established and maintained by the direct and indirect actions of both the miRNA and the TF, each promoting a gene expression profile unique to the cell state in which they are expressed. Further, as miRNA and TF networks are notoriously complicated to unravel, this work offers a novel approach to disentangling such complexity, combining cell line perturbations with gene regulatory network analysis and multi-factor linear modelling to tease apart and make sense of the complex and layered networks of gene regulation.

## Materials and methods

### Cell culture

The MDA-MB-231 (ATCC: HTB-26) immortalised human breast cancer cell line was obtained from American Type Culture Collection (ATCC, Rockville, MD) and used to generate ZEB-expressing stable cell lines. All MDA-MB-231 derived cell lines were cultured in Dulbecco’s Modified Eagle Medium (DMEM; Gibco) supplemented with 10% fetal bovine serum (Invitrogen). Cells were sub-cultured every 2–3 days, using 0.2% Trypsin/1× PBS to dissociate cells during passaging. All cell lines tested negative for mycoplasma.

### Stable cell generation

To generate cell lines with inducible ZEB1 and ZEB2 expression, pENTR2B-ZEB2 (HsCD00829572, Centre for Personalized Diagnostics, DNASU) was recombined with pInducer21 using LR Clonase to produce pInducer21-ZEB2 [15, 16]. GFP is independently encoded within the same vector as ZEB2 and is driven by the same promoter though is not a fusion protein. pInducer20-ZEB1 was previously generated by PCR and restriction enzyme cloning [17]. Plasmids were transformed into competent *E. coli* cells, purified by QIAfilter Plasmid Midi Kit (#12243, Qiagen) and sanger sequenced for insert verification.

For lentivirus production, HEK293T cells were plated at 2 × 10^6^ cells in T25 flasks. The following day, cells were transfected with pLP1, pLP2, pLP-VSVG, pTAT (1 µg of each) and 4 µg of either pInducer20-ZEB1 or pInducer21-ZEB2. DNA was mixed in 500 µl opti-MEM (Invitrogen) and transfected in combination with 12 µl Lipofectamine-2000 (Invitrogen). Transfection reagent was removed 6hrs post transfection and viral supernatant of either pInducer20-ZEB1 and pInducer21-ZEB2 was collected after 72 h. MDA-MB-231 cells were seeded at 2 × 10^5^ cells in T25 flasks. The following day, cells were co-transduced with viral supernatant of pInducer20-ZEB1 and pInducer21-ZEB2 (1:8 each) in the presence of polybrene (4 mg/ml). Polyclonal or single cell selection was done using 1 mg/ml of G418, followed by cell sorting for GFP expression (encoded on pInducer21-ZEB2 vector).

Cells were sorted by GFP fluorescence intensity compared to control cells (parental MDA-MB-231, no colour). Cell sorting was performed on the MoFlo Astrios EQ High Speed Cell Sorter using Summit Software version: 6.3.1 (Beckman Coulter, Miami, FL, USA). Single clones and pools were selected for strength of ZEB1 and ZEB2 induction by qPCR and western blotting, ensuring endogenous-like and supraphysiological levels of expression following miR-200c transfection. In all instances, miR-200c refers to the predominant guide strand, miR-200c-3p.

### Transfection

Cells were seeded at 2 × 10^5^ cells per well in 6-well plates and treated with or without 0.5 µg/mL Doxycycline (DOX) to induce expression of ZEB1 and ZEB2. The following day, cells were transfected with 5nM miR-200c mimic (GenePharma) or 5nM negative control RNA, using optiMEM (Invitrogen) and Lipofectamine RNAiMAX (Invitrogen) as per manufacturers protocol. Media was replaced 6 h post transfection. DOX was replenished in fresh media every 48 h.

### RNA isolation, cDNA synthesis and qRT-PCR

Total RNA was harvested 72 h post transfection using TRIzol (Invitrogen), following standard manufacturers protocol. Complementary DNA was synthesised from 500ng RNA using the QuantiTech Reverse Transcription Kit (Qiagen). Quantitative RT-PCR was performed on a Rotor-Gene 6000 series thermocycler (Qiagen) with Quantitect SYBR Green reagent (Qiagen). Analysis was performed using the comparative quantitation feature in the Rotor-Gene software with each gene measured being normalized to the mean of GAPDH and ß-Actin. All qRT-PCR primers are listed in Supplementary Table 1.

### RNA-sequencing

High quality RNA samples (in biological duplicate from parental, endogZEB and oexZEB cells + 5nM negative control or miR-200c miRNA mimic (genepharma)) were DNAse treated according to manufacturers protocol (#AM1907, TURBO DNA-f*ree*™ Kit) prior to library preparation. Total RNA was treated with RNAseH and strand-specific libraries were prepared and sequenced using a single-end 75 cycle high output kit on an Illumina NextSeq500 at the ACRF Cancer Genomics Facility. The FASTQ files were analyzed and quality-checked using FastQC v0.11.9 [18]. Reads were mapped against the human reference genome (build hg38) using the STAR (v2.6.1d) spliced alignment algorithm [19] with parameters --twopassMode basic and --quantMode GeneCounts and otherwise default parameters.

### Linear modelling of gene expression

Linear modelling of differential gene expression was performed using limma [20]. Genes were filtered to discard lowly expressed genes (≥ 1 Count Per Million (CPM) in at least 2 samples). Libraries were normalised using the “TMM” method and principal component analysis was performed (Supplementary Fig. 1). This identified a small but consistent batch effect associated with biological replicate in PC6 (~ 2% of variance); batch was therefore included as a covariate in linear modelling. Linear modelling was performed incorporating the variables of cell background (Parental, endogZEB or oexZEB), miR treatment (miR-200c or control miR transfection) and technical replicate batch (1 or 2) and dox treatment (with or without dox treatment).

Importantly, these variables were combined in 2 ways. For clarity, design matrixes for both models are included in Supplementary Fig. 2A. Firstly, a single variable model was fitted, where the miR treatment (“control miRNA” or “miR-200”) and cell line ZEB level (“Parental”, “Endogenous-level ZEB”, “Overexpression ZEB”) were combined into a single categorical variable (e.g. with values “control miRNA in Parental cells”, “miR-200 in Parental cells”, “control miRNA in Endogenous-level ZEB cells”, “miR-200 in Endogenous-level ZEB cells”, “control miRNA in Overexpression ZEB cells”, “miR-200 in Overexpression ZEB cells”).

Independently, a second model, termed the ‘interaction’ model was fitted by including two variables, one for miR treatment (“control miRNA” or “miR-200”) and one for cell line ZEB level (“Parental”, “Endogenous-level ZEB”, “Overexpression ZEB”). To account for differences in the miR response in different cell lines, two interaction terms were included (to capture the “endogenousZEB: miR” and “overexpressionZEB: miR” interactions).

The limma voom function [21] was used to calculate precision weights and the limma lmFit, eBayes and topTable functions were used to fit, refine and evaluate the models. Genes with a q-value of ≤ 0.05 were considered significant. As positive controls, the fit of the models was examined for several genes known to be regulated by ZEB or miR-200 by plotting the fitted coefficients against the CPM values (examples shown for CDH1 and CFL2 in Supplementary Fig. 2B) and by examining the sigma values of overall model fit. We observed generally excellent fits for the models.

For heatmaps of gene expression levels, batch effects relating to biological replicate were removed using the limma::removeBatchEffect function which retained effects relating to the ZEB cell line background, miR transfection type and dox treatment. Log_2_CPM values for each gene were then centred by their mid-point (the average of the gene’s maximum and minimum expression) and scaled by their standard deviation.

### Exon intron split analysis (EISA)

EISA [22] was performed as described in [12]. Briefly, we used only non-overlapping genes and uniquely mapped reads to quantify the number of reads in intronic and exonic regions in a strand-specific manner for all UCSC RefSeq mRNA transcripts from each gene. For each contrast analysed using limma above, EISA was performed in a strand-aware manner (eisa_general_script.R with flag --choose-strand r1). Results were combined and CPM normalisation was performed separately for intronic and exonic read counts.

### MicroRNA targeting prediction

Predicted targets of the human miR-200c were obtained from files downloaded from Targetscan (v7.2) (miR name: hsa-miR-200c, Homo sapiens species code: 9606, files: “miR_family_Info.txt”, “Summary_Counts.all_predictions.txt”) [23]. Additional target prediction (Fig. 2H) was obtained from Diana microT-CDS (ver5) [23], miRGeneDB (ver 2.1) [24] and PicTar [25].

### Gene correlation network analysis

Weighted gene correlation network analysis was performed using the R package WGCNA [26]. Log2 CPM gene expression data (6 groups, 12 samples) was used, excluding the control samples with no-dox treatment and discarding noncoding gene classes with names starting with “SNOR”, “SCARN”, “RNU”, “MIR” due to being inaccurately quantified in this library type. Biological replicate batch effects were removed using limma::removeBatchEffect as described above and expressed genes were selected by requiring expression of ≥ 4 CPM in at least 2 samples. Genes were ranked to select the top 3000 most differentially expressed genes (according to the difference between their second-highest and second-lowest expression values). This threshold was found to capture many known miR-200 and ZEB target genes.

A signed network of samples was constructed using the adjacency function with distOptions= “method=‘euclidean’”. The data was raised to a soft power of 16 for calculation of the signed adjacency matrix and a tree was constructed from the signed topological dissimilarity matrix using method=“average”. To select gene clusters, we performed dynamic branch cutting (method=“hybrid”, minClusterSize = 10, deepSplitParam = 0) and merged closely related modules (“salmon” became “brown”; “greenyellow” became “cyan”, “pink” became “black”, “tan” became “blue”). For better visualisation, “Light cyan” was relabelled “pink” and “light yellow” was relabelled “orange” (original and final clusters shown in Supplementary Fig. 3A). Module eigengenes were determined. Module membership, a generalised version of intramodular connectivity, was calculated for each gene and module eigengene combination, defined as the correlation between the two. ZEB and miR Gene Significance was also calculated, defined as the correlation between the miR or ZEB level category and the gene expression. Small modules comprising < 50 genes were discarded (“lightgreen”, “orange”, “grey60”, “royalblue”). The resulting network was exported using the exportNetworktoCytoscape function (with weighted = TRUE, threshold = 0.1) visualised in Gephi [27].

### Integrative bioinformatics analyses

Visualising linear modelling results grouped by gene correlation network models. Violin plots of the expression levels of genes in each module relative to the Parental control group (“Parental CTRL”) (Supplementary Fig. 3C) were produced by plotting the fitted coefficients from linear modelling after first mean-centring the data for each gene and then normalising to the expression in the Parental CTRL group. For the violin plot of the fitted coefficients for the interaction linear model per gene module (Fig. 5B), the coefficient values were plotted raw.

The heatmap (Fig. 5C) integrating data from many sources was prepared using the ComplexHeatmap R package [28] as follows: genes were ordered by module according to their position in the network graph diagram. Within a module, genes were ordered according to their intramodular connectivity with most highly connected genes in the centre and less-connected genes lying left or right based on ratio of their connectivity with the two adjacent modules. To indicate modules with strong positive or negative association with miR or ZEB expression level, ZEB and miR Gene Significance correlation values calculated above were plotted. ZEB1 ChIP-seq data was obtained from ENCODE (HepG2 cells; GSM1010809; GM12878 cells; GSM803411) and MDA-MB-231 cells [29]. “2 out of 3 ZEB1 ChIP-Seq” denotes common genes bound by ZEB1 in at least 2 of the 3 datasets. “Top 252 ZEB1 ChIP-Seq” denotes genes bound by ZEB1 in MDA-MB-231 cells and in the top 50% of peak scores in at least one of HepG2 and/or GM12878 cells (252 genes were identified by such analysis). TargetScan scores used “Total context + + score” values, displaying only values lower than − 0.2. The statistical significance of the enrichment of ChIP or miR targets within modules was evaluated using Fisher’s Exact Tests. Linear modelling (“exonic”) and EISA (“intronic” and “exon-intron”) output was plotted as log_2_ fold-changes.

### Gene ontology analyses

For each gene module, gene ontology analysis was performed using geneontology.org. The 377 Gene Ontology terms were manually grouped into broad categories (“Cytoskeleton”, “Adhesion”, “Transcription”, etc.). The columns “Direct_targets”, “EPI” and “MES” indicate whether the biological term was also enriched in lists of : Direct targets (miR-200c TargetScan score prediction <−0.2), epithelial (“EPI”) genes or mesenchymal (“MES”) genes (Ontologies derived from lists of genes 5 fold upregulated in HMLE cells compared to mesHMLE cells (“EPI”, 406 genes) or 4 fold upregulated in mesHMLE cells compared to HMLE cells (“MES”, 418 genes)).

### Protein purification and Western blotting

For preparation of protein lysates, cells were collected after 72 h, washed with ice cold 1× PBS and lysed with 1x RIPA lysis buffer (Abcam) containing cOmplete Mini, EDTA-free Protease-inhibitor (Roche) and PhosSTOP phosphatase inhibitor (Roche). The concentration of protein in purified lysate was estimated using the Pierce BCA Protein Assay Kit (Thermo Scientific). 20 µg of protein was loaded onto Bolt Bis–Tris Plus 4–10% gradient gels using 1x Bolt MOPS SDS Running Buffer (Invitrogen). Proteins were transferred to nitrocellulose membrane at 4 °C using 1x Bolt Transfer Buffer (Invitrogen) with 10% methanol by volume. Membranes were incubated with Ponceau stain for total protein visualization using ChemidocTouch. Membranes were blocked in 5% skim milk for 1 h at RT and incubated overnight in primary antibody (ZEB1, 1:1000, #D3013, SantaCruz Bio; E-Cadherin, 1:1000, #610182, BD Biosciences Pharmigen) at 4 °C. Protein visualization using Near Infrared (NIR) was achieved by incubation for an hour at 4 °C in secondary (1:10,000) antibody, IRDye 800, of the correct species. The same membrane was re-probed with α-tubulin (1:10,000, #ab7291, Abcam) for an hour at 4 °C followed by an hour of secondary (1:20,000) antibody, IRDye 680.

### Migration

Migration was assessed by both transwell migration assay (toward a chemo-attractant) and scratch assay (undirected migration on a plastic surface). For transwell migration, following DOX induction and miR-200c transfection, cells were seeded (2 × 10^5^ cells) without serum in Transwells (8µM pore size; Costar) covered in complete media. Cells were then incubated at 37 °C for 4 h before fixing in formalin (LABCHEM Neutral Buffered formalin, 2518–10 L PL). Transwells were washed with PBS, permeabilized using 0.1% Triton X-100, stained with DAPI (400ng/ml in methanol), and mounted with DAKO fluorescence mounting medium (Agilent). Cells were imaged by fluorescence microscopy using the 10x objective lens, counted using ImageJ and averaged across six fields x 4 technical replicates per condition. For scratch assay, a cell-free area was cleared using a pipette tip 1 day post miRNA transfection. Cells were photographed 24 and 48 h later. All assays were performed in biological triplicate.

### Cell proliferation

Transfected cells were plated in 96 well plates at 3 × 10^3^ cells per well, 24 h post- DOX treatment and miR-200c/NC transfection. Cells were incubated with Incucyte Nuclight Rapid Red Dye (#4717, Sartorius) according to manufacturers instruction prior to reading the first cell number using the Incucyte Live-Cell Analysis System (Sartorius). Live cells were imaged every 4 h for 96 h. Growth curves and doubling times were calculated by labelled cell number metrics.

### Gelatin degradation

Coverslips (18 mm diameter) were prepared by subjecting to a 30% nitric acid wash (30 min) followed by 0.01% poly-L-lysine (Sigma-Aldrich) coating (15 min) and crosslinking with 0.5% glutaraldehyde (10 min, including extensive PBS washing). Gelatin coating was performed by incubating coverslips with prewarmed 0.1% Oregon Green 488-conjugated gelatin (Invitrogen) and 0.2% porcine gelatin for 10 min to which 5 mg/ml NaBH4 was added (3 min). Coverslips were incubated in complete media for 2 h at 37 °C before seeding cells (2 × 10^4^ cells/ml) and overnight incubation. Cells were then fixed with 4% paraformaldehyde in 5% sucrose (20 min at 37 °C).

### Cell adhesion

Parental, endogZEB and oexZEB cells were treated with Dox (0.5ug/ml). One day later, cells were transfected with control or miR-200c mimics, and plated with fresh Dox into 24 well trays that were uncoated, or pre-coated with either fibronectin or a 50/50 combination of fibronectin and Matrigel. 2 days later, cells were continuously shaken for 15 min, media removed, washed once with PBS, fixed by the addition of 4% paraformaldehyde for 10 min, washed again with PBS and stained with crystal violet (1%w/v in 2% ethanol) for 10 min. Crystal violet was removed and washed 5x with water. Trays were then dried and adherent cells imaged (Oprtonix GelCount Colony Counter, Oxford). Quantitation of crystal violet was performed by solubilisation in 10% acetic acid and measurement at 570nM (FLUOstar Omega plate reader, BMG Labtech). Anchorage-independent growth.

Parental, endogZEB and oexZEB cells were treated with Dox (0.5ug/ml). One day later, cells were transfected with control or miR-200c mimics and plated onto ultra-low adhesion plates (Corning). Cells were imaged 3 days later.

### Immunofluorescence

Morphology was assessed by plating 5 × 10^4^ cells onto 13 mm fibronectin coated glass coverslips in a 24 well plate. Coverslips were fixed 72 h post-transfection in 4% paraformaldehyde for 10 min at room temperature (RT), then washed with 1x PBS. Cells were permeabilized in 0.1% Triton X-100-PBS (PBT) for 5 min followed by blocking in 1% bovine serum albumin (BSA, Bovogen) for 1 h at RT. Coverslips were then immunolabelled with ZO-1 (1:100 overnight at 4 °C; D6L1E, #13663, Cell Signalling Technologies), followed by appropriate secondary antibodies and AlexaFluor 647-Phalloidin (1:1000 for 30 min at RT, Invitrogen). Cells fixed on gelatin coverslips were permeabilized in 0.5% PBT (5 min), prior to immunolabelling with Cortactin (1:100 overnight at 4 °C, (#05–180, Millipore) and AlexaFluor 647-Phalloidin (1:300 for 1 h at RT). All coverslips were mounted in Vectashield mounting media (VectorLabs). Imaging was performed using Zeiss LSM 700 and Leica SP8 confocal microscopes. Quantitation of gelatin degradation was performed counting a minimum of 200 cells per variable. All assays were performed in biological triplicate.

### Data and code availability

Raw RNA-seq data and gene counts have been deposited to the NCBI’s Gene Expression Omnibus and are accessible through GEO Series accession GSE289003. Source code for the workflows and analyses presented here are available in the repository at https://bitbucket.org/sacgf/sourdin_supertargets_2025.

## Results

### The miR-200 family and the ZEB transcription factors constitute a prominent negative feedback loop

**Fig. 1:**
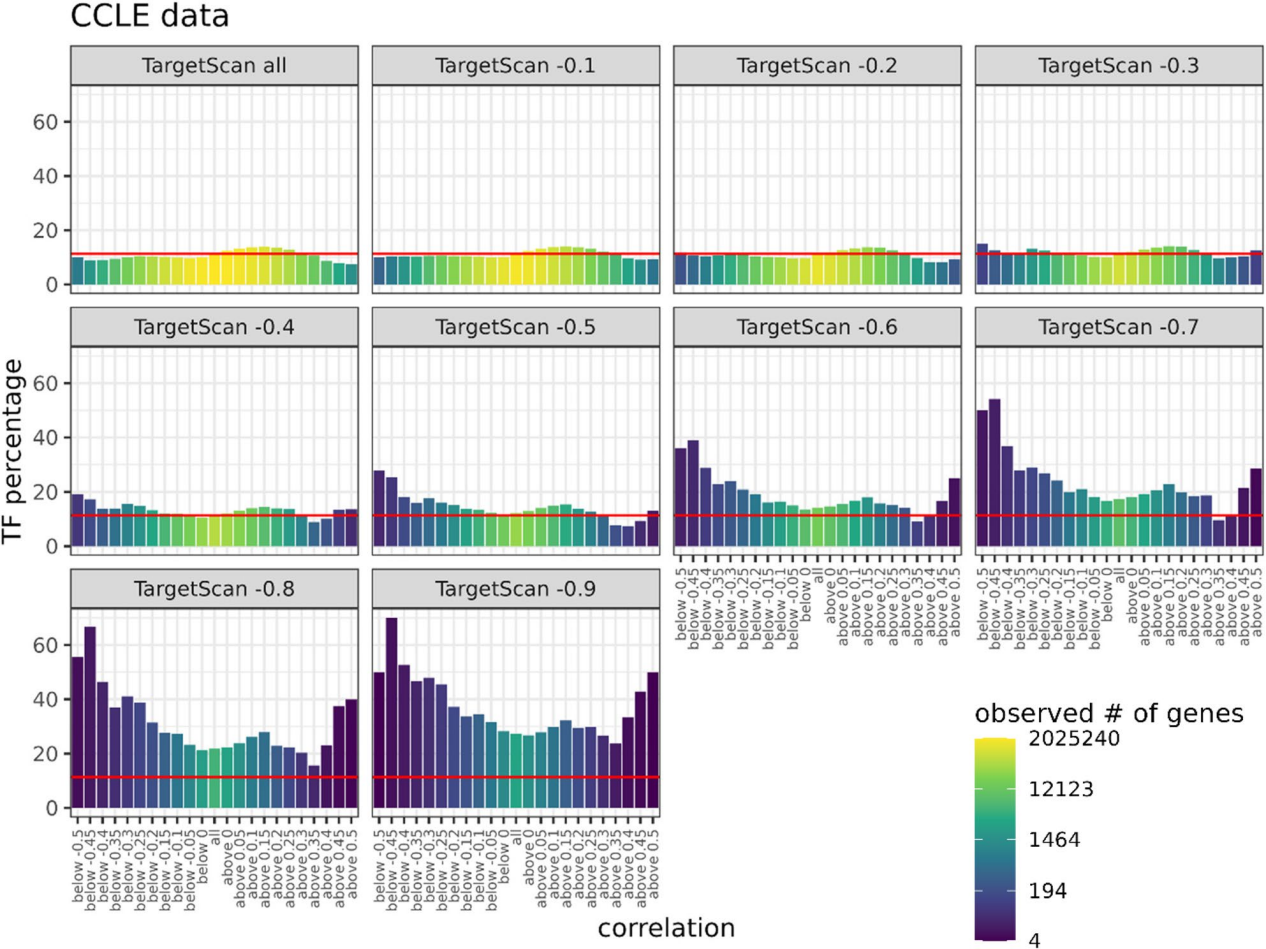
Transcription factors are disproportionately highly represented among miRNA targets. From among the top 550 most confidently annotated miRNAs, putative target genes were grouped by the strength of their predicted targeting (Targetscan scores >−0.1 to >−0.9, the more negative the number the greater the predicted targeting strength) and the degree of correlated expression between the miRNA and target across the CCLE (~ 1000 cell lines). Colouring represents the numbers of targets. Bar height represents the proportion of targets that are transcription factors. The red line represents the proportion of transcription factors that are expected given their genomic representation. Supplementary Table 2 lists the specific numbers of genes associated with each graph

We rationalise that the most important miRNA target genes are likely to be strongly-regulated TFs as the effect of their suppression will permeate throughout gene expression networks. In an effort to assess the abundance of TFs among miRNA target genes, we assessed both the confidence with which miRNA targets were predicted and the correlation of expression between miRNAs and mRNAs across the Cancer Cell Line Encyclopedia (CCLE, ~ 1000 cell lines). We found TFs to be progressively more enriched among increasingly confidently predicted target genes and that for such targets, there was an increasing tendency for the expression the TF and the miRNA to be correlated, either negatively or positively (Fig. 1, Supplementary Table 2). This implies not only are TFs more likely to be strongly targeted by miRNAs compared to other types of genes, but that cross-regulation, either direct or indirect, between miRNAs and TFs is abundant. This observation is broadly consistent with previous reports that TFs are over-represented within miRNA regulatory networks [4, 6, 7] and includes the seemingly unexpected observation that in addition to negatively correlated expression between a miRNA and its target (consistent with canonical miRNA function and the establishment of mutually exclusive bistable cell states), target genes can also be positively correlated with the targeting miRNA [30]. This is likely the result of additional regulators acting in feed-forward gene regulatory motifs and a point to a buffering role for the miRNA in limiting TF expression beyond a certain threshold.

Due to the prevalence of TFs among prominent miRNA targets and the potential for dual negative feedback to establish and maintain separate cell states, we sought to create a workable definition of “strong” miRNA targets” and identify the most prominent examples for further investigation. Of the > 9 million miRNA - target gene predictions in Targetscan, and > 11 million miRNA - gene pairs whose relative expression was correlated across the CCLE, only 337 were present in both the top 1% of most confidently predicted miRNA targets (Targetscan) and the top 1% of negatively correlated expression (CCLE). In agreement with Figs. 1 and 84 of the 337 genes (25%) were TFs - well above their expected level of ~ 12% in the human protein coding genome. The top 3 miRNA - target pairs were all the ZEB1 TF, paired with various members of the miR-200 family (Supplementary Table 3). When the list of “strong” targets were expanded to the top 5% of Targetscan predictions and CCLE expression correlation, 2028 miRNA - target gene pairs were identified, including all combinations of both ZEB1 and ZEB2 with each of the 5 members of the miR-200 family (Supplementary Table 3).

**Fig. 2:**
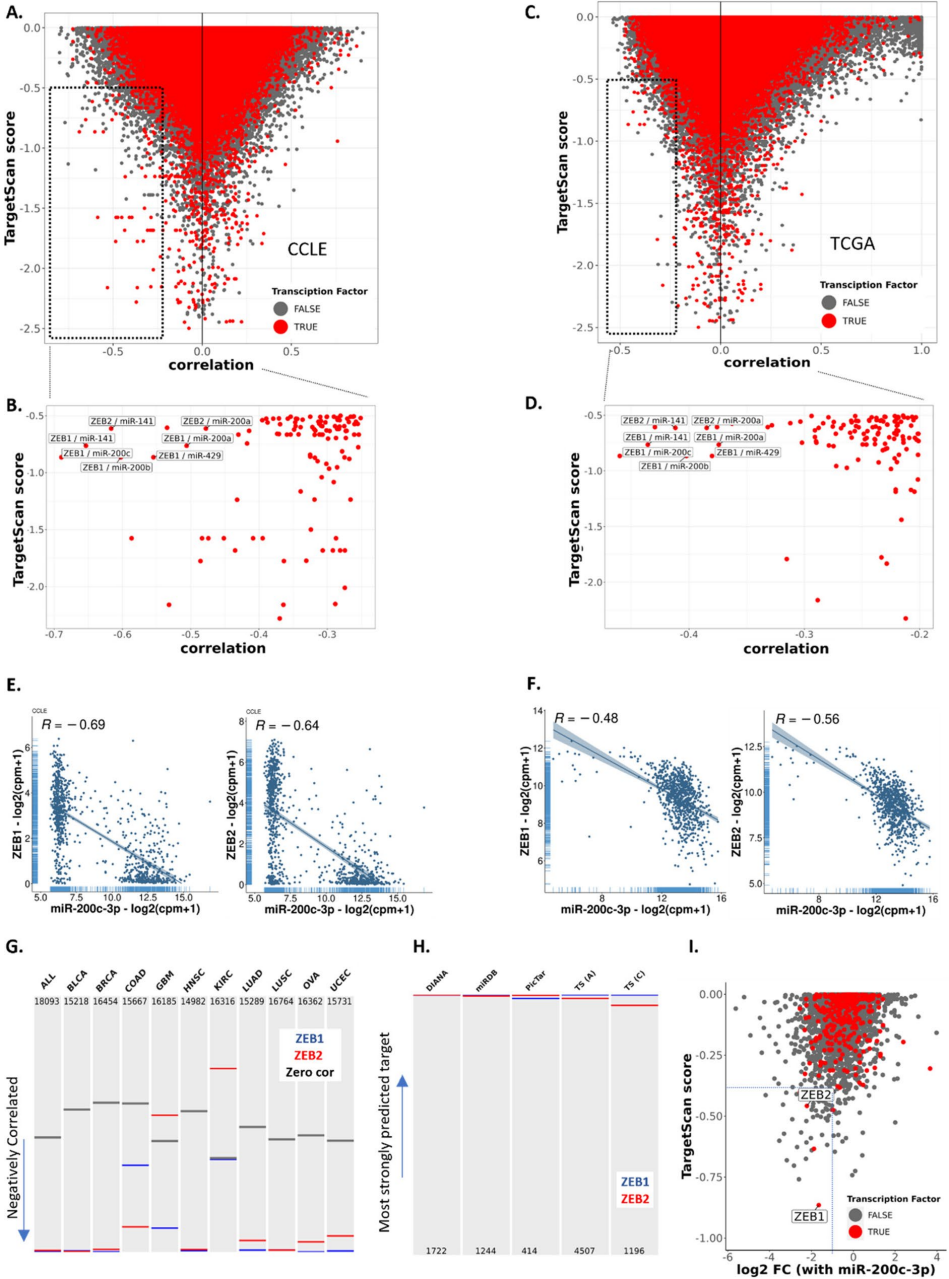
The miR-200:ZEB axis is prominent among putative microRNA: target gene pairs **A**-**D** Strength of miRNA target prediction (TargetScan score; more negative = more strongly predicted) and correlation of miRNA: putative target expression (REC score) across the CCLE or breast cancer TCGA. Transcription factors (foreground) are represented as red dots, all other genes as grey dots. Boxed section is enlarged in B, D. The 550 highest confidence miRNAs are included in this analysis **E**-**F** Correlation of ZEB and miR-200c expression across CCLE (E) and TCGA (breast cancer) (F) **G** Ranking of correlated expression from 10 cancer types across TCGA between ZEB1 (blue) or ZEB2 (red) and miR-200c. Genes are ranked from most to least correlated with horizontal lines indicating the ranked position of correlation with miR-200c relative to all other genes. The position of zero correlation (grey bar) is indicated **H** Ranked targeting strength of all putative miR-200c target genes as predicted by multiple algorithms. Number of targets predicted by each algorithm are shown at the bottom **I** Targetscan score and log fold change of all genes 3-days post miR-200c transfection in MDA-MB-231 cells. Transcription factors are again indicated by red dots. All other genes are represented as grey dots. The specific position of ZEB1 and ZEB2 are indicated. Dotted line indicates the 5th percentiles of the most strongly predicted targets and of genes downregulated in response to miR-200c

We therefore sought to examine the notion of prominent miRNA - TF targeting through the lens of miR-200 and ZEB (Fig. 2A-D); key components of an established reciprocal feedback loop that governs the epithelial or mesenchymal state of cells [13, 14, 31, 32]. Further examination revealed strongly negatively correlated expression between miR-200c and ZEB family members across both cell lines and cancer patients (Fig. 2E, F). This is true across most cancer types (glioblastoma and kidney renal cell carcinoma being exceptions, Fig. 2G). Further, from among the thousands of genes whose 3’UTRs are predicted to be bound by miR-200, ZEB1 and ZEB2 rank among those most confidently predicted across of series of target prediction algorithms (Fig. 2H). Lastly, primacy of the miR-200c: ZEB relationship was supported experimentally, wherein ZEB1 and ZEB2 were among only a handful of genes ranked in both the top 5th percentiles for both target prediction strength and transcript down-regulation after miR-200c expression (Fig. 2I) For these reasons, we hypothesized that the miR-200:ZEB axis could be used as a model to further investigate the influence of strongly regulated TFs within miRNA-regulated networks and to model the complexities of network regulation where a miRNA-TF axis has evolved to mediate bistable cell states.

### Targeting of ZEB is responsible for much of the transcriptomic effect of miR-200, including Mesenchymal-Epithelial transition (MET)

**Fig. 3:**
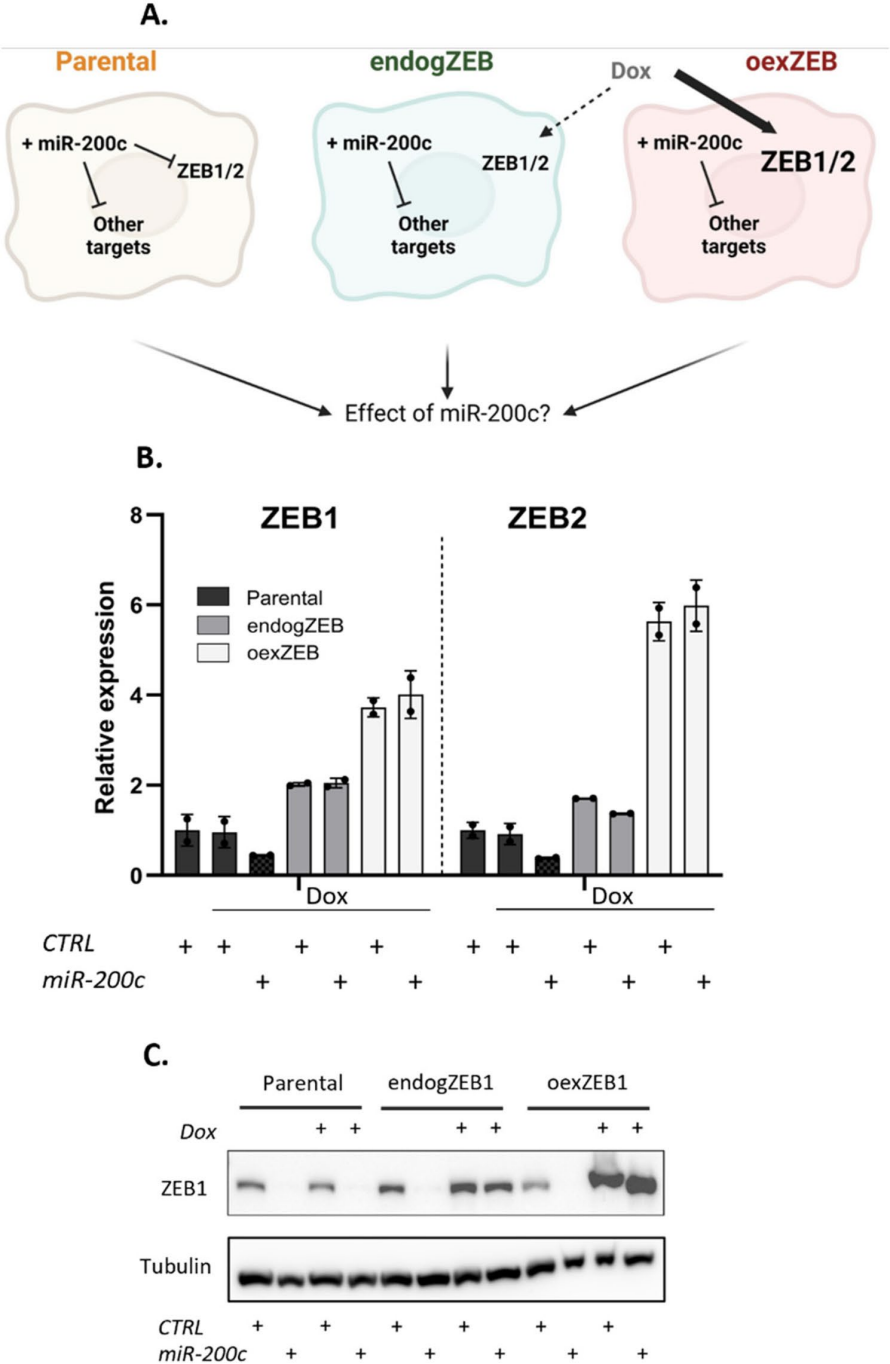
Establishment of a cell system to test ZEB-dependency in the response to miR-200c. **A** Outline of stable ZEB expression established in MDA-MB-231 cells. In endogZEB and oexZEB cell lines, stably incorporated, dox-inducible ZEB1 and ZEB2 (lacking 3’UTRs) have been introduced to maintain (endogZEB) or over-express (oexZEB) both ZEB1 and ZEB2, even in the presence of exogenous miR-200c. **B** ZEB1 and ZEB2 RNA expression as determined from whole-cell sequencing. **C** ZEB1 protein expression, +/- Dox across stable cell lines

To examine the extent to which strong TF targeting is responsible for the function of a miRNA with thousands of candidate target genes, we created stable MDA-MB-231 cells expressing ZEB1 and ZEB2 (lacking 3’UTRs and thus without the capacity for the miR-200 family to downregulate their expression) (Fig. 3A). In one cell line designed to emulate endogenous ZEB expression levels (“endogZEB”), upon Dox induction ZEB was maintained at levels consistent with that expressed physiologically. By comparing the different response between these cells and the parental line, the specific impact of the loss of ZEB targeting can be seen. A second cell line was also created which emulated the over-expression of ZEB (“oexZEB”), wherein both ZEBs are intentionally expressed at levels above which they are normally present. This cell line can be used to determine which of the effects of miR-200 that ZEB is capable of over-riding (Fig. 3B, C).

**Fig. 4:**
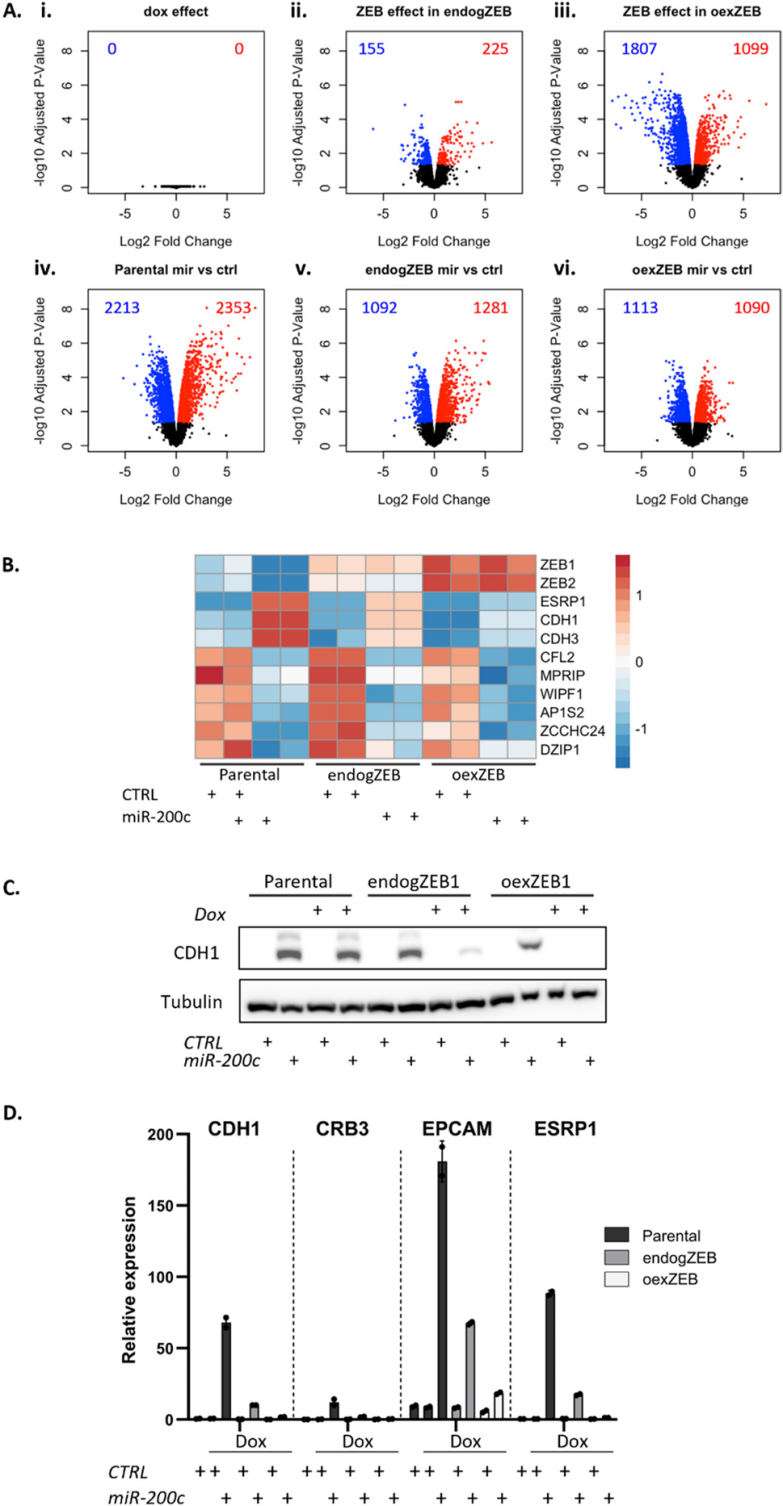
Repression of ZEB is essential for miR-200c to promote epithelial gene expression. **A** The influence of ZEB and/or miR-200c is represented by log2 fold change vs. expression graphs for all genes as determined by whole cell sequencing. Statistically significant changes (FDR < 0.05) are indicated by coloured dots. **B** Heat map indicating changes in the expression of ZEB1 and ZEB2, and established direct ZEB1 (ESRP1, CDH1, CDH3) and miR-200c (CFL2, MPRIP, WIPF1, AP1S2, ZCCHC24 and DZIP1) target genes. **C** E-cadherin protein expression and **D** expression of key epithelial markers as determined by RNA-seq, with or without Dox across the stable cell lines. As they were derived from the same experiment, anti-Tubulin blots are equivalent for Figs. 3c and 4c

Seeking to determine the quality of the experimental system and data, differential expression and multidimentional scaling (MDS) analyses demonstrate that dox itself has no effect in parental cells and that the variation between biological replicates is small (Fig. 4Ai, Supplementary Fig. 1). As expected, maintenance of ZEB expression has minimal effects (4Aii), whilst over-expression leads to an extensive range of transcriptomic alterations (4Aiii). Further analysis indicates miR-200c drives a series of indirect effects, as the genes that are affected by miR-200c expression are as likely to be upregulated (indirectly) as they are to be downregulated (either directly or indirectly) (4Aiv). The impact of miR-200c was diminished in endogZEB and oexZEB cells relative to parental cells, both in terms of number of genes affected and the magnitude of the gene expression change (4Av and vi compared to iv), suggesting many of miR-200c’s largest transcriptomic effects are ZEB-dependent.

Looking in specific detail at established miR-200c direct target genes (CFL2, MPRIP, WIPF1, AP1S2, ZCCHC24, DZIP1), these were consistently downregulated by miR-200c irrespective of ZEB level (Fig. 4B). Also as anticipated, endogenous ZEB was only downregulated by miR-200c in parental cells, with the endogenous and elevated levels of expression in endogZEB and oexZEB cells otherwise unaffected by miR-200c. Most striking however were the effects on a range of epithelial markers (CDH1, CDH3, CRB3, EPCAM, ESRP1) – typically used as the primary readout for the epithelial state of cells. In each case, dramatically elevated gene expression in response to miR-200c in parental cells was largely eliminated under conditions of ZEB restoration (endogZEB) and completely blocked by elevated ZEB expression (oexZEB) (Fig. 4B-D, Supplementary Figs. 4). Similar results were seen in BT549 cells in which Dox-inducible ZEB was also stably incorporated (Supplementary Fig. 5) and in mesHMLE cells transiently transfected with both ZEB expression vectors and miR-200c (Supplementary Fig. 6). This indicates that the capacity for miR-200c to enforce a core epithelial gene expression program (its primary function in literature) is essentially completely dependent upon its capacity to target ZEB. Whilst this may appear surprising given that target prediction algorithms identify in excess of 4,000 putative miR-200c target genes, the unusually large quantity and quality of miR-200 target sites within ZEB 3’UTRs (Fig. 1H) also suggests the targeting of ZEBs are especially prominent.

### The miR-200 : ZEB axis controls a plethora of gene regulatory events at multiple levels

**Fig. 5:**
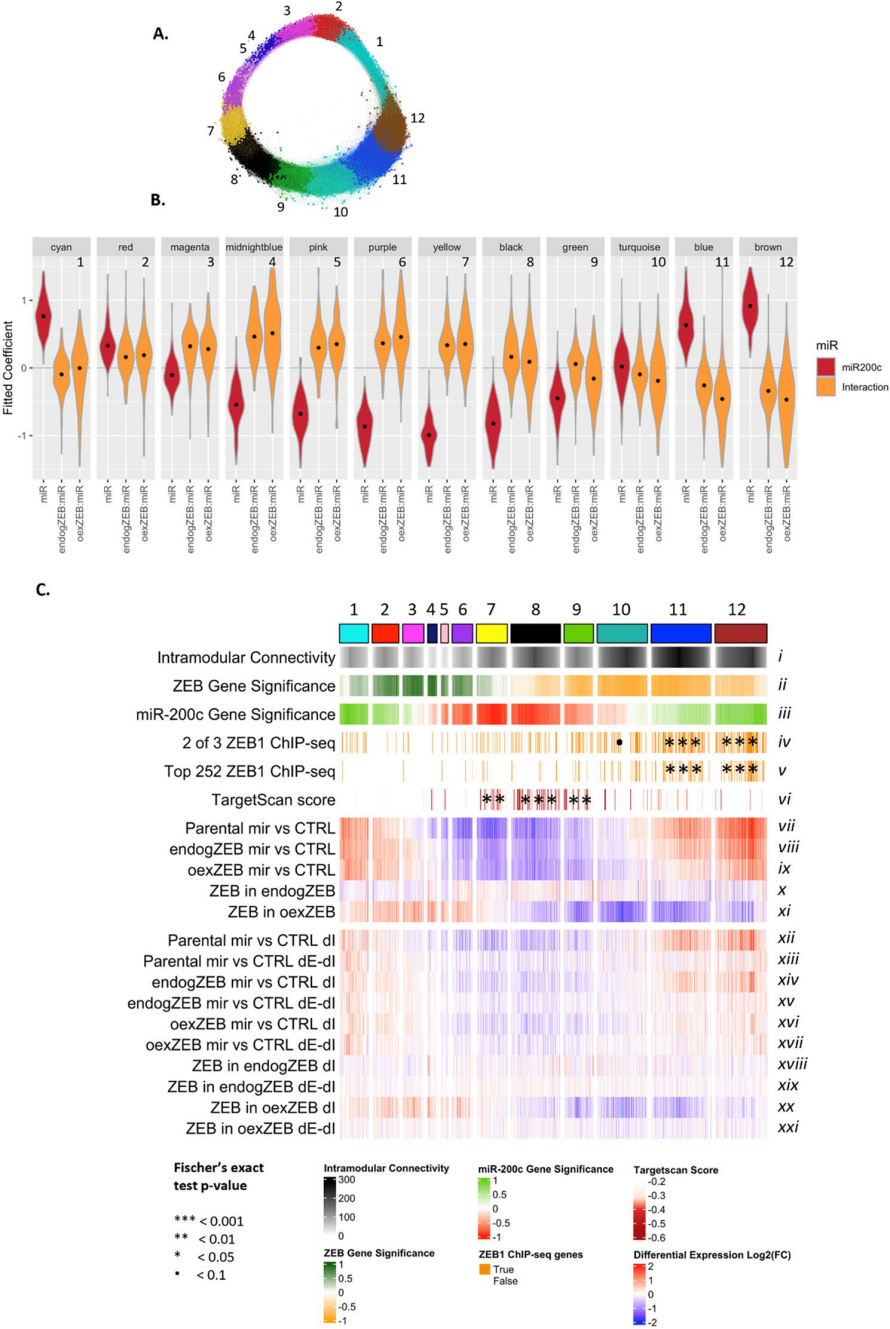
Gene-specific responses to miR-200c expression in the presence and absence of ZEB1 and ZEB2. **A** A weighted gene co-expression network was constructed in which genes were assigned to one of 12 modules (indicated by different colours), based upon the similarity of their gene expression profile. **B** The interaction between the influence of the miRNA and the presence of varying levels of ZEB is shown by modelling gene expression changes using a fitted co-efficient model for each of the gene modules. Any capacity of endogZEB or oexZEP to modulate the effect of miR-200c is indicated by orange violin plots above or below the 0 coefficient. miR-200c-responsive gene expression changes in parental cells are indicated by red violin plots. **C** The most highly responsive 5000 genes are organised into their gene modules and cross-referenced with further details relevant to the expression of that gene. Individual genes are indicated by vertical lines. Rows (ii) and (iii) indicate correlation of gene expression with ZEB1 and miR-200c expression respectively. Rows (iv) and (v) highlight if the promoter of the gene in question was bound by ZEB1 in 2 or the 3 ZEB1 ChIP-seq datasets (iv) or if the promoter was bound by ZEB1 in MDA-MB-231 cells and was among the top 50% of enriched peaks in either HepG2 or GM12878 cells (v). Predicted strength of direct miR-200c targeting (Targetscan score) is shown in (vi). The magnitude of gene expression change between the comparisons indicated are shown in (vii – xi). The degree to which the gene expression change occurs at the transcriptional (ΔI) or post-transcriptional (ΔE-ΔI) level is determined by EISA (xii – xxi). Roman numerals have been assigned to each category to improve clarity of discussion. Statistical significance of gene enrichment within modules is calculated by Fisher’s exact t-test

Although the effects on key epithelial markers or established miR-200 target genes are clear, and likely biologically important, they still represent only a tiny proportion of genes that are responsive to miR-200c. Correlation network analyses are powerful methods to reveal the global regulatory structure in datasets, providing insights which would be missed by simple pair-wise differential expression analyses. To characterise the miRNA and ZEB regulatory relationships, a weighted gene co-expression network analysis was conducted using WGCNA, which used similarity between gene expressions to assign regulated genes to twelve colour-coded clusters (or “modules”) (Fig. 5A, Supplementary Fig. 3A). Unexpectedly but intriguingly, a graph visualisation of the network revealed a clear circular structure, completely unlike the clumped, “hairball”-style structures usually observed for this method. To evaluate whether the circular nature was an artifact of the soft power data transformation step, we performed PCA analysis of WGCNA network both with and without this transformation (i.e. soft power of 16 or 1). The results for dimensions 1 and 2 (Supplementary Fig. 3B) confirmed unequivocally that the circular structure in our analysis (left) was not caused by the soft power data transformation step as the untransformed data (right) produced an even more classically circular shape, comprising an even larger share of the total variance (> 99% vs. ~ 70%).

Combining linear modelling with gene correlation network analysis, by clustered the linear modelling coefficients according to the gene correlation network modules, we determined the direction and strength of regulation by the miR-200 and ZEB for each cluster. Presented as a violin plot (Fig. 5B), positive values indicate the factor (ZEB in orange or miR-200 in red) acts as an activator on these genes, whereas negative values indicate the factor acts as a repressor. The wave-like pattern (Fig. 5B) revealed that the circular shape of the network graph (Fig. 5A) is driven by the underpinning mutually repressive feedback loop between the miRNA and ZEB factors; this causes the regulatory network to be a single continuum of degrees of activation and repression by the two factors.

Examining which modules contained known gene targets of miR-200c and ZEB revealed clear regions of the circular structure of particular interest. The black (#8) module contained numerous validated miR-200c targets, including CFL2, AP1S2, WIPF1, QKI and ZCCHC24. Genes assigned to this module were prominently downregulated by miR-200c in a ZEB-independent manner (indicated in Fig. 5B by an average fitted coefficient in response to miR-200c of ~−0.8 Log_2_FC; shown in red, and ~ 0 Log_2_FC for the same genes in endogZEB or oexZEB cells; shown in orange). This indicates the presence of ZEB does little to further modulate the response to the miRNA. Similarly, the blue (#11) and brown (#12) modules contained many crucial epithelial genes (CDH1/3, ESRP1/2, CRB3) that are known to be directly bound and repressed by ZEB. Consistent with this, genes in this module displayed prominent upregulation in response to miR-200c (red) which was abrogated by ZEB expression (orange).

Genes associated with turquoise (#10), red (#2) and magenta (#3) modules, were affected by miR-200c to a much smaller magnitude than genes in other modules (Fig. 5B, Supplementary Fig. 3C). Amongst the complex series of gene regulation, it is notable that although there are groups of genes that are responsive to miR-200 but are unaffected by ZEB (or vice versa), with the exception of the red module in which the magnitude of the gene expression changes are very small, there are no modules for which the effect of miR-200 and ZEB moves gene expression in the same direction (for example, a gene upregulated in response to miR-200c and further upregulated upon ZEB expression). Consistent with reciprocal feedback and the establishment of mutually exclusive cell states, this indicates miR-200c and ZEB, likely through a range of both direct and indirect mechanisms, oppose gene expression driven by the other axis component.

To further investigate mechanisms of regulation, Exon-Intron Split Analysis (EISA) was applied; a bioinformatic technique wherein contrasting differences in read coverage in intronic vs. exonic regions can reveal whether genes are regulated at transcriptional or post-transcriptional levels [22]. The underlying premise is that intronic reads are derived from nascent, unspliced pre-mRNA and thus changes in intronic read coverage (ΔI) reflect transcriptional regulation. By this logic, changes in exonic read coverage (ΔE) is derived from the sum of transcriptional and post-transcriptional regulation; in practice this means post-transcriptional regulation can then be deduced by subtracting the transcriptional (intronic) changes (ΔI) from the exonic ones (ΔE) (further details provided in Materials and Methods). The successful application of EISA is indicated by results for two sets of control genes: known direct post-transcriptional targets of miR-200c show a decrease in ΔE-ΔI, whilst established transcriptional targets of ZEB repression become de-repressed in response to miR-200c-mediated ZEB knockdown, exclusively at the level of ΔI (Supplementary Fig. 7).

The participation of miRNAs within transcriptional networks is complex, as in addition to the strong targeting of a small subset of transcripts to exert biological function, a plethora of modest regulation is also exerted across the transcriptome which can minimise genetic noise and dampen (buffer) the effects of transient stimuli. In addition to revealing examples of prominent transcriptional and post-transcriptional regulation, EISA also reveals global buffering between transcriptional and post-transcriptional regulatory arms as the genes that are down-regulated post-transcriptionally are more likely to be transcriptionally upregulated and vice versa (Supplementary Fig. 8). This is consistent with the miR-200:ZEB axis operating within a complex network of gene expression that involves the activities of many factors beyond the direct effect of miR-200c itself, and with a need for stability in the expression of many genes, even during an EMT/MET switch. It also reflects that whilst the most prominent individual miRNA: target interactions drive biological effects, a large number of miRNA interactions are associated with the buffering of gene expression and transcriptomic noise [33]. However, multiple genes are also the subject of dual regulation by both miR-200 and ZEB as indicated by high-confidence target prediction, high-throughput miR-200:target co-precipitation and the presence of ZEB1-ChIP peaks within target gene promoters (Supplementary Fig. 9). Such regulatory modules represent composite coherent and incoherent feed-forward loops where the complementary or opposing actions of miR-200 and ZEB either serve to drive mesenchymal-specific gene expression (coherent), or to promote homeostatic buffering (incoherent); this has been associated previously with hybrid EMT cell states [34–36].

Gene correlation analyses between miR-200c and ZEB demonstrated a continuum of regulation hypothesised to underpin the circular network graph structure (Fig. 5A). To infer miR-200 and ZEB direct and indirect mechanisms of gene regulation for each of the gene modules, we built evidence of the regulatory circuits underpinning these modules through integration with ChIP-seq and Targetscan prediction and EISA data (Fig. 5C). Direct ZEB target genes were identified through the analysis of three ZEB-1 ChIP-Seq studies [29, 37, 38], including one performed in the same cell line (MDA-MB-231) in which genes were further denoted as targets for activation or repression after ZEB1 expression [29]. For example, genes in the green (#9), black (#8), yellow (#7) and purple (#6) modules are downregulated by miR-200c in a largely ZEB-independent manner (indicated by a similar degree of blue intensity between the parental, endogZEB and oexZEB cells; compare *vii – ix*). At least for green (#9), black (#8) and yellow (#7) modules, there is an enrichment in genes predicted to be directly targeted by miR-200c (*vi*) and accordingly, these modules contain many well established direct miR-200c target genes (CFL2, AP1S2, SEC23A etc.). Interestingly however, even here a substantial amount of the gene expression changes across the modules are occurring at the transcriptional level (evidenced in the high colour intensity of ΔI compared to the ΔE-ΔI rows; *xii - xvii*). This further indicates the complex landscape of gene regulation where even among a group of genes that are enriched in direct miR-200c targets, a significant proportion of their expression is co-ordinately controlled through transcriptional means, both dependent (black #8 and green #9) and independent (yellow #7) of ZEB.

The blue (#11) and brown (#12) modules comprise additional sets of strongly regulated genes. Here, miR-200c-driven changes are also transcriptional, but in this case genes are upregulated in a ZEB-dependent manner (red intensity decreases indicating dampening of the miR-200 effect at increasingly high levels of ZEB; compare *vii – ix* and *xii* with *xiv* and *xvi*). Accordingly, ChIP-seq analyses showed the brown and blue modules exhibit the highest enrichment of ZEB1 binding to promoters (*iv*,* v*).

### The miR-200:ZEB axis co-ordinates gene regulation via multiple mechanisms

**Fig. 6:**
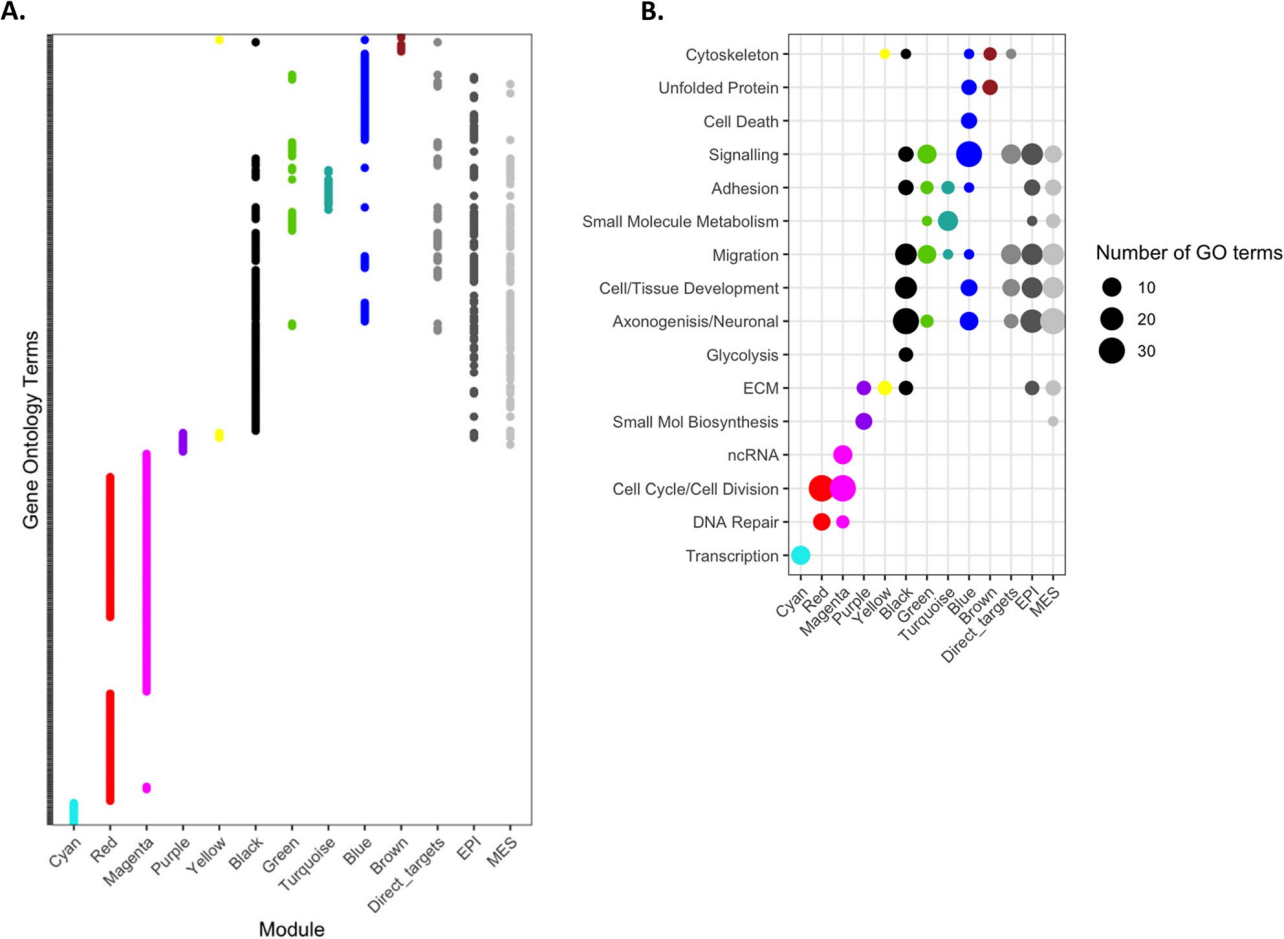
Common EMT-associated processes are responsive to manipulation of the miR-200:ZEB axis, despite differences in the mechanisms of gene regulation. Gene ontologies of miR-200:ZEB responsive genes as assigned to different WCGNA (Fig. 5) expression modules. Enriched ontologies (left panel) are grouped (right panel) into more generalised processes. Specific ontology enrichments are shown in Supplementary Table 4

EMT is associated with alterations in cell signalling pathways and regulation of the cytoskeleton, the extracellular matrix and cell adhesion that culminates in changes to the migratory capacity of cells [39]. Gene ontology analysis reveals that modules clustered in different regions of the circular network graph are associated with different biological processes (Fig. 6, Supplementary Table 4). Genes in the yellow (#7) module for example, enriched in direct miR-200c targets, are associated with the extracellular matrix and cytoskeleton, whilst genes associated with other aspects of EMT (including migration, signalling, development and neurogenesis) are regulated through combinations of direct miR-200c (green #9, black #8) and ZEB (turquoise #10, blue #11) targeting.

Interesting alternative regulatory patterns associated with smaller numbers of genes are demonstrated by the cyan (#1), red (#2) and magenta (#3) modules. These genes show non-canonical regulation patterns, such as upregulation in response to miR-200c in the cyan (#1) module (likely due to indirect effects mediated by other TFs) or upregulation by ZEB in a largely miR-200c-independent manner (magenta, #3). Associated with these modules are an enrichment of non-coding RNAs, and genes that participate in transcription, DNA repair and cell division. These same processes are not differentially enriched between epithelial and mesenchymal states (Fig. 6B), nor are they generally associated with EMT. However, the magnitude of gene expression changes within these modules is small (Fig. 5B, Supplementary Fig. 3C) and though there are many sub-ontologies that fall within these categories which are enriched (Fig. 6A), the modest degree of gene regulation likely lessens the impact of these effects. Collectively, these data indicate that the complex network of differential gene expression between epithelial and mesenchymal states is opposingly driven via the miR-200:ZEB axis, promoting bi-stability through reciprocal negative feedback and via additional indirect mechanisms.

### miR-200 controls a range of phenotypic responses that are both ZEB-dependent and ZEB-independent

**Fig. 7:**
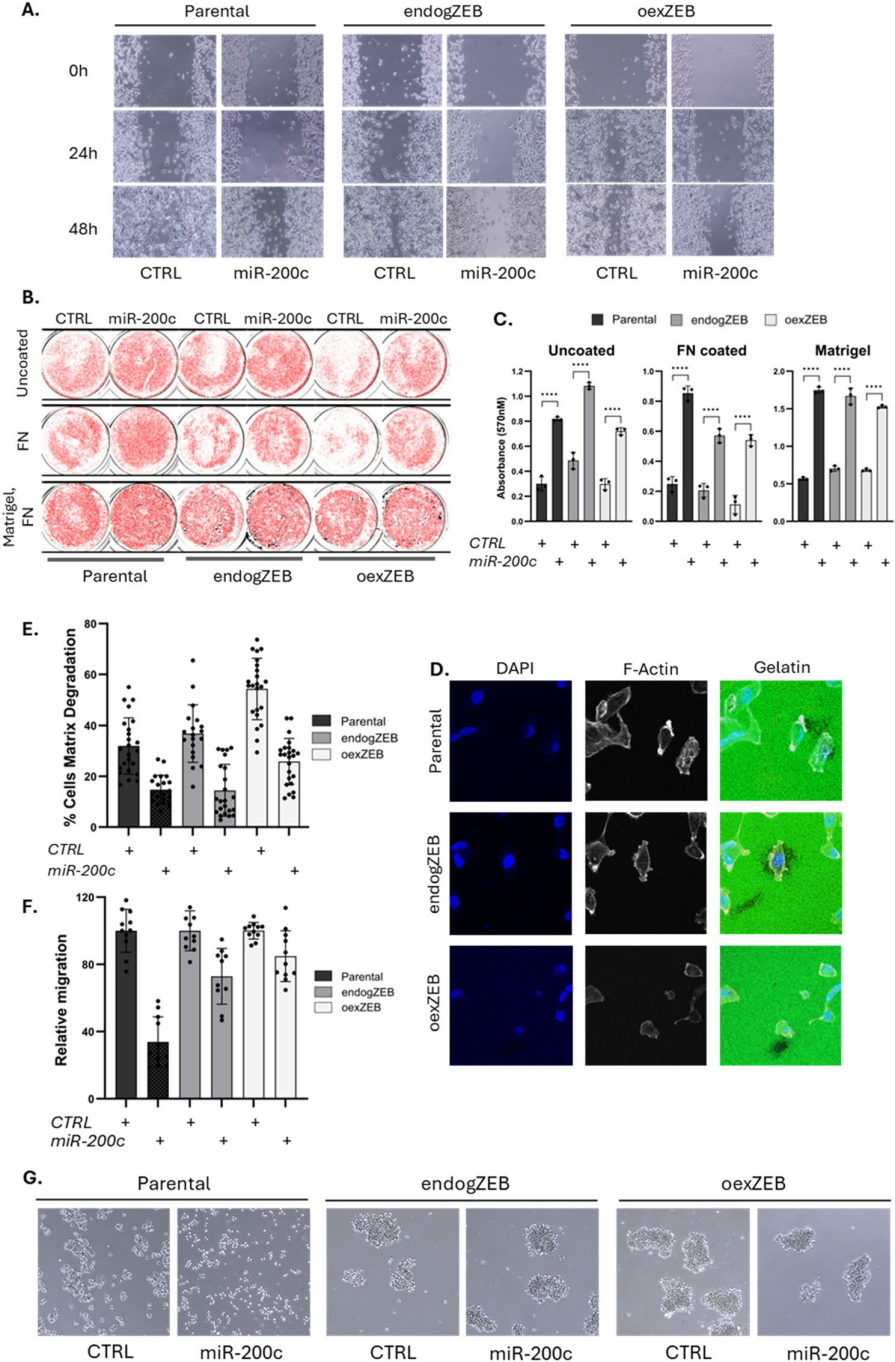
Variable ZEB-dependency in the phenotypic response to miR-200c. Functional assays were performed in MDA-MB-231 stable cell lines transfected with control or miR-200c miRNA mimic one day after ZEB-induction via Dox. **A** Migration across an uncoated surface was assessed via scratch assay, visualised 24–48 h post-scratch. **B**, **C**) Crystal violet staining (B) and quantitation (C) of MDA-MB-231 stable cell lines remaining adhered to uncoated tissue culture dishes, or dishes pre-coated with fibronectin or 50/50 matrigel + fibronectin after 15 min shaking. Each dot represents a biological replicate. **D** Nuclei (DAPI, blue), actin (phalloidin, red) and degradation of a fluorescently labelled gelatin matrix (green) were assessed and representative images shown. **E** Percentages of cells for which matrix degradation is visible. A minimum of 250 cells were assessed in each sample. **F** Cell migration toward a chemoattractant was determined via transwell migration assay. Each dot represents a biological replicate. **G** Anchorage-independent growth and survival was assessed by growing cells in suspension in ultra-low adhesion plates. Representative images are shown 3 days after transfection. Significance is calculated by unpaired t-test against the control; * *p* = < 0.05; ** *p* = < 0.01; *** *p* = < 0.001

It is well established that at least in some model systems, disruption of the miR-200:ZEB feedback loop affects “classical” EMT properties including cell migration, invasion and adhesion. We therefore sought to establish which phenotypic effects resulting from miR-200c perturbation are ZEB-dependent. In the case of cell migration as assessed by scratch assay (Fig. 7A), miR-200c inhibited migration irrespective of ZEB expression. MiR-200c also promoted the adhesion of cells regardless of the substrate to which they are attached (Fig. 7B, C), and suppressed the capacity of cells to degrade a gelatin matrix upon which they are growing - a result of less invadopodia - again both independently of ZEB (Fig. 7D, E). Thus, although the upregulation of key epithelial markers are largely a result of miR-200c suppressing ZEB (Fig. 4B-D, Supplementary Fig. 4–6), this alone does not explain all of the phenotypic effects of miR-200c perturbation. Such observations are likely the result of a host of cytoskeletal genes that are enriched among direct miR-200c targets [40]. On the other hand, miR-200c suppressing the migration of cells toward a chemoattractant is largely eliminated by ZEB (Fig. 7F), as is the clustering of non-adherent MDA-MB-231 cells (grown in ultra-low adhesion plates) which is suppressed by miR-200c, but only when ZEB is not maintained (Fig. 7F). In the MDA-MB-231 system, miR-200 did not affect proliferation (Supplementary Fig. 10A), consistent with cell cycle associated genes being solely enriched within gene modules characterised by minimal gene expression changes in response to miR-200c (red #2, magenta #3; Fig. 5B). Similarly, neither cell morphology (Supplementary Fig. 10B), nor the rate of EGFR internalisation and recycling (Supplementary Fig. 10C, D) were affected by miR-200c. Collectively, these data suggest that miR-200 has the capacity to affect a wide range of cellular processes through a combination of ZEB-dependent and ZEB-independent mechanisms, an observation consistent with broad transcriptomic effects of which some are dependent upon ZEB and others not.

## Discussion

A major objective in miRNA research is to understand how specific targeting events translate into phenotypic outcomes. This task is complicated by the nature of miRNA–mRNA interactions: short seed sequences enable individual miRNAs to potentially regulate thousands of transcripts. As a result, target prediction algorithms often yield high false-positive rates [41], and high-throughput biochemical methods that map miRNA-binding sites en masse offer little guidance on which interactions are biologically significant. Most miRNA-target interactions are weak, resulting in modest repression [42, 43], so the phenotypic consequence of any single targeting event is usually minimal, even though the cumulative impact of miRNA activity can be substantial [44–46].

Amid this vast regulatory landscape, TFs stand out as particularly consequential targets. By modulating the expression of TFs, miRNAs can exert far-reaching effects that reverberate through gene regulatory networks. Consistent with this, transcriptome-wide effects of miRNA perturbation are often dominated by transcriptional rather than direct post-transcriptional changes [11, 12]. TFs are also disproportionally highly represented among miRNA targets. For example, 25% of miR-124 targets are TFs even though TFs only represent ~ 10% of genes [47]. Here, we find that TFs are progressively more represented among increasingly strongly predicted targets (Fig. 1), indicating that not only are TFs frequently targeted, but that this targeting is likely to be particularly meaningful. Multiple lines of evidence also suggest that reciprocal regulatory loops between miRNAs and TFs are over-represented in gene regulatory networks [10, 48]. Extending these links further, miRNAs are more likely to co-target TFs that physically interact [5], miRNA target genes are often enriched among the regulons of miRNA-targeting TFs [49], and feedback involving miRNAs are common features of TF autoregulatory loops [50].

Among regulatory architectures, mutual negative feedback loops - where a miRNA and a TF repress each other - are especially intriguing. These circuits are ideally suited to function as bistable switches, toggling cells between discrete phenotypic states. Such motifs are crucial during differentiation and, when dysregulated, are implicated in cancer progression [51, 52]. Documented examples include reciprocal repression between YY1 and miR-1/miR-29 in skeletal myogenesis [53, 54], NFI-A and miR-223 in granulopoiesis [55], Pitx3 and miR-133b in dopaminergic neurons [56], and the central focus of this study: the mutual antagonism between the miR-200 family and ZEB transcription factors, which governs EMT [13, 14, 31, 32].

Given the importance and abundance of this type of regulatory axis, we looked to dissect a prominent example by establishing an experimental system that decouples ZEB repression from the broader regulatory activity of miR-200c, for the first time enabling the specific consequences of ZEB targeting to be assessed independently of other miR-200 targets. In this system, miR-200c retains its full targeting repertoire, with the exception of ZEB. Strikingly, when ZEB repression is blocked, miR-200c loses its ability to induce epithelial gene expression and greatly diminishes its capacity to inhibit motility, indicating a large requirement for the silencing of ZEB in order for miR-200c to fulfil its core EMT-inhibitory functions. Having said this, we also note ZEB-independent functions such as degradation of a gelatin matrix (a readout of invadeopodia) which is suppressed by miR-200c whether or not the expression of the ZEBs are maintained. This likely occurs via direct repression of cytoskeletal and matrix metalloprotease genes [40, 57] (Fig. 6).

A central reason for the intense interest in EMT/MET lies in its role promoting metastasis. “Classical” EMT traits, such as loss of epithelial adhesion and acquisition of motility and invasion, are well-documented contributors to metastatic dissemination. In our MDA-MB-231 model, we observe phenotypes that encompass both ZEB-dependent effects of miR-200c (transwell migration, epithelial gene expression), and ZEB-independent features (invadopodia formation, cell adhesion). However, it is now widely recognised that EMT extends beyond these classical traits. Increasingly appreciated “non-classical” roles include the regulation of stemness, resistance to apoptosis and therapy, metabolic reprogramming, and immune evasion [34, 58]. Moreover, EMT is no longer regarded as a binary switch between discrete epithelial and mesenchymal states. Instead, multiple intermediate or hybrid E/M states exist, with cells maintaining an epigenetic memory that allows them to maintain features consistent with their history, even in the context of other phenotypic changes [58]. EMT is therefore a multifaceted and context-dependent process. As such, no single miRNA-target gene relationship can be expected to account for the full spectrum of EMT-associated phenotypes, nor can findings in one cellular system be assumed to apply universally.

This study is therefore limited by the specific cellular context employed. With the exception of epithelial marker expression assessed in two additional cell lines (Supplementary Figs. 5 and 6), the transcriptomic and functional analyses were conducted in MDA-MB-231 cells, a mesenchymal-like breast cancer model that does not respond to all EMT/MET cues. Consequently, there are “non-classical” characteristics of EMT we have not examined in the context of miR-200 - ZEB dependency. Further, just because certain aspects of miR-200 function are ZEB-dependent, this does not necessarily mean that other strongly targeted TFs also dominate the function of other miRNAs, though our work, coupled with wider analysis of transcriptomic data after miRNA perturbation [11, 12], suggests this property is likely widespread beyond the miR-200 – ZEB axis.

**Fig. 8:**
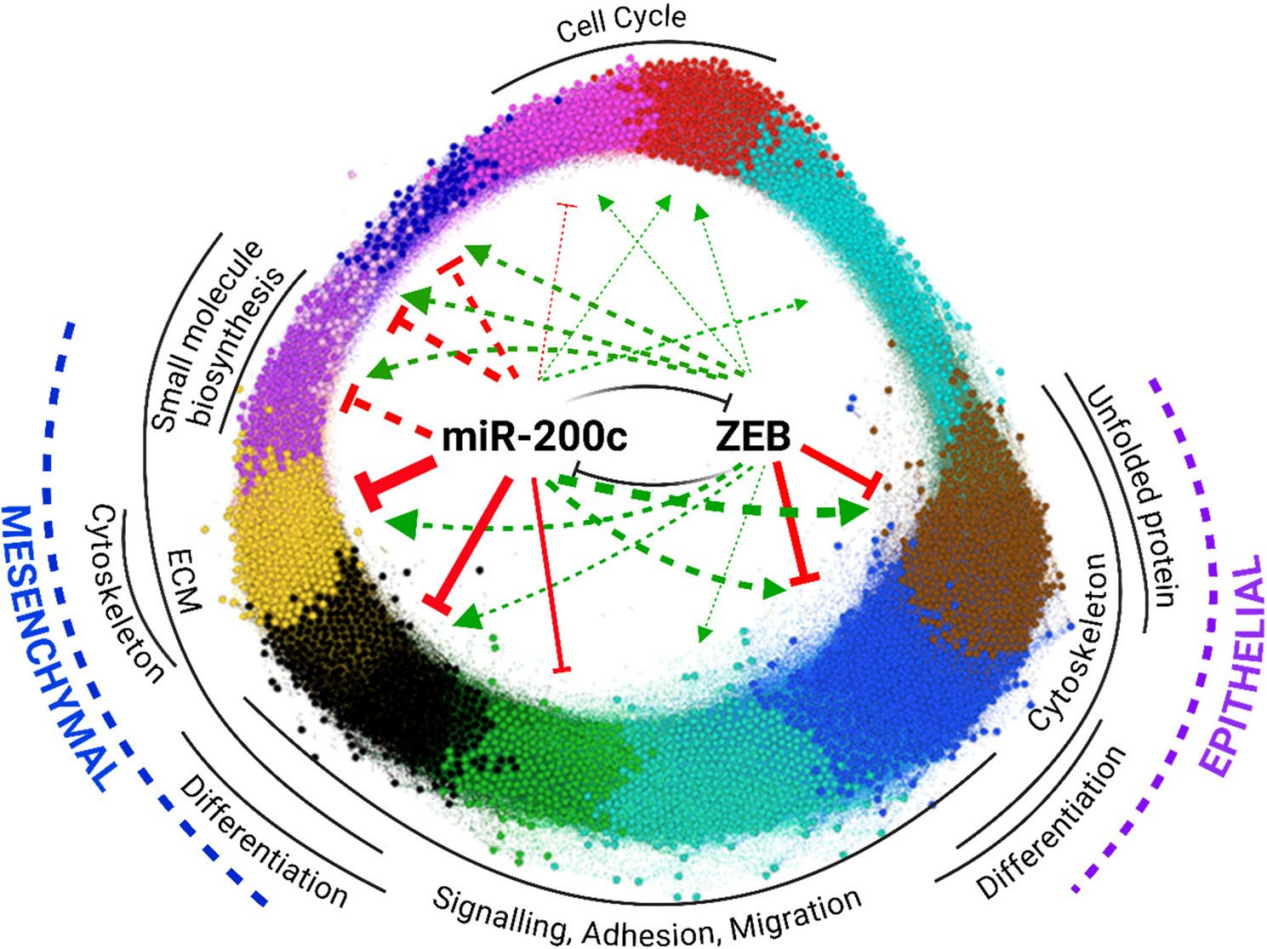
Gene modules regulated by the miR-200 : ZEB feedback loop. WGCNA-derived gene module regulation by miR-200c and ZEB as derived from linear modelling (Fig. 5b). The mean strength of regulation is reflected by line thickness. Predominantly directly regulated modules are indicated by unbroken lines

Several conclusions emerge from this work. First, TFs constitute a special class of miRNA targets, becoming increasingly enriched among the target pool as predicted targeting efficiency increases. This suggests evolutionary selection for miRNA-TF interactions as a core mechanism for modulating transcriptional outputs. Indeed, expression of highly predicted TF targets can be either positively or negatively correlated with their regulating miRNAs, consistent with two dominant regulatory logics: toggle switches (mutual repression) and buffering circuits (where miRNAs limit over-activation of TFs controlled by shared upstream stimuli, Fig. 1). Second, we find that although there are major ZEB-independent effects that contribute to miR-200c driven MET related to cell invasion and adhesion (Fig. 7A-E), epithelial gene expression - the most commonly used measure of MET/EMT - is strongly ZEB-dependent (Fig. 4C, D, Supplementary Figs. 4–6). This argues that at least in some important aspects of miRNA function, the primacy of select TF targets is important. Perhaps most revealing of all however, integrated systems-level analyses - combining linear modelling, WGCNA, and EISA - reveal a highly complex continuum of gene expression states following miR-200c induction, involving extensive direct and indirect effects of miR-200c and ZEB that reinforce mutually exclusive expression programs involving EMT-relevant genes (Figs. 5 and 8). An example of the extent of these indirect effects is that even among the top 250 most post-transcriptionally downregulated genes upon miR-200c expression, only 17% contain canonical miR-200 binding sites. One would assume additional epithelial-enforcing miRNAs, themselves indirectly upregulated by miR-200c expression, likely contribute to this observation [59]. Gene regulatory networks are also characterised by feedforward loops in which a miRNA and TF jointly regulate shared target genes. Hundreds of such loops have been implicated in cancer [60, 61], and similar relationships are evident in our data, including co-regulation of TFs such as FLI1 and TFAP2A by both miR-200c and ZEB (Supplementary Fig. 9).

Whilst this work characterises the complex nature of the miR-200 : ZEB regulatory axis, a broader question is raised regarding the abundance of other miRNA-TF axes. Using similarly stringent thresholds across the CCLE dataset, we identified several high-confidence feedback pairs, including known examples (let-7 and ARID3 [62], miR-29 and TET [63]) and novel candidates (e.g., miR-29a and ZBTB34, miR-29a and ZKSCAN4, miR-766 and GLIS2, miR-19 and BNC2, miR-210 and LYL1) (Supplementary Table 3). When prediction thresholds are slightly relaxed, dozens more candidate feedback loops emerge. These findings, together with other established regulatory systems (let-7/HMGA2/LIN28B [64, 65], YY1/miR-1/−9 [53, 54], NFIA/miR-223 [55], Pitx3/miR-133b [56]) support the hypothesis that strong miRNA-TF regulatory loops are widespread and may drive many of the transcriptional changes observed following miRNA perturbation [11, 12].

While we focused here on the miRNA-mediated repression of ZEB, the reciprocal regulation of miR-200 by ZEB has also been examined from the opposite perspective where CRISPR has been used to delete the ZEB binding sites in the miR-200c/−141 promoter. Though the analysis of this data did not include the systems approach applied here, it was interesting that the dynamics of TGFβ-induced EMT was altered from a hysteretic to linear transition, thus highlighting the importance of tight feedback for bistability. Although both hysteretic and non-hysteretic EMT states show similar invasiveness and morphology, only hysteretic transitions enhance metastatic colonization in vivo [66], underscoring the clinical relevance of feedback architecture.

In summary, we show that bistable cell fate decisions can be reinforced through multilayered regulatory logic involving direct and indirect, transcriptional and post-transcriptional interactions between miRNAs and TFs that affect the expression of thousands of genes. In the case of miR-200c, we show that expression of a core set of genes typically used to define the epithelial state, as well as other transcriptomic effects regulated by miR-200c, are indeed dependent upon the repression of ZEB. This provides proof of concept that a small number of TF “super-targets” may dominate miRNA-driven regulatory responses, though we note that even in the context of one of the strongest of all miRNA – target gene axes (Supplementary Table 3), processes such as cell adhesion and invadeopodial formation are affected by miR-200c in a ZEB-independent manner, meaning that a wider network of miR-200 targets are also vital. The complex interplay we describe here likely extends beyond the miR-200 : ZEB axis, representing a generalizable systems principle of how miRNAs orchestrate robust cellular transitions. Our approach, combining targeted perturbations, transcriptomic profiling, and network-level modelling, provides a powerful framework for dissecting the complexity of gene regulatory circuits.

## Supplementary Information

Supplementary Material 1.

## Data Availability

Raw RNA-seq data and gene counts have been deposited to the NCBI’s Gene Expression Omnibus and are accessible through GEO Series accession [GSE289003](https:/www.ncbi.nlm.nih.gov/geo/query/acc.cgi acc=GSE289003). Source code for the workflows and analyses presented here are available in the repository at https://bitbucket.org/sacgf/sourdin\_supertargets\_2025.

## Abbreviations

AGO: Argonaute
CCLE: Cancer cell line encyclopedia
EISA: Exon-intron split analysis
EMT: Epithelial-mesenchymal transition
MET: Mesenchymal-epithelial transition
miR: MicroRNA
RISC: RNA-induced silencing complex
TCGA: The cancer genome atlas
TF: Transcription factor
UTR: Untranslated region
