# Supplementary material for: Strongly regulated transcription factors exert an outsized influence in microRNA-regulated networks
Source: Cell Commun Signal. 2025 Dec 27;24:63. doi: 10.1186/s12964-025-02626-w (PMC12853912; doi:10.1186/s12964-025-02626-w)
Supplement: Supplementary file 1 — Supplementary Material 1. [file 12964_2025_2626_MOESM1_ESM.pdf]

Supplementary Data

Strongly regulated transcription factors exert an outsized influence in microRNA-regulated networks

Laura Sourdin[1], Julie M Bracken[1], Philip A Gregory[1,2], Nora Feldker[3,4], Thomas Brabletz[3], Simone Brabletz[3], Yeesim Khew-Goodall[1,2,5], Gregory J Goodall[1,2,5], Katherine A Pillman*[1,6] and Cameron P Bracken*[1,5]

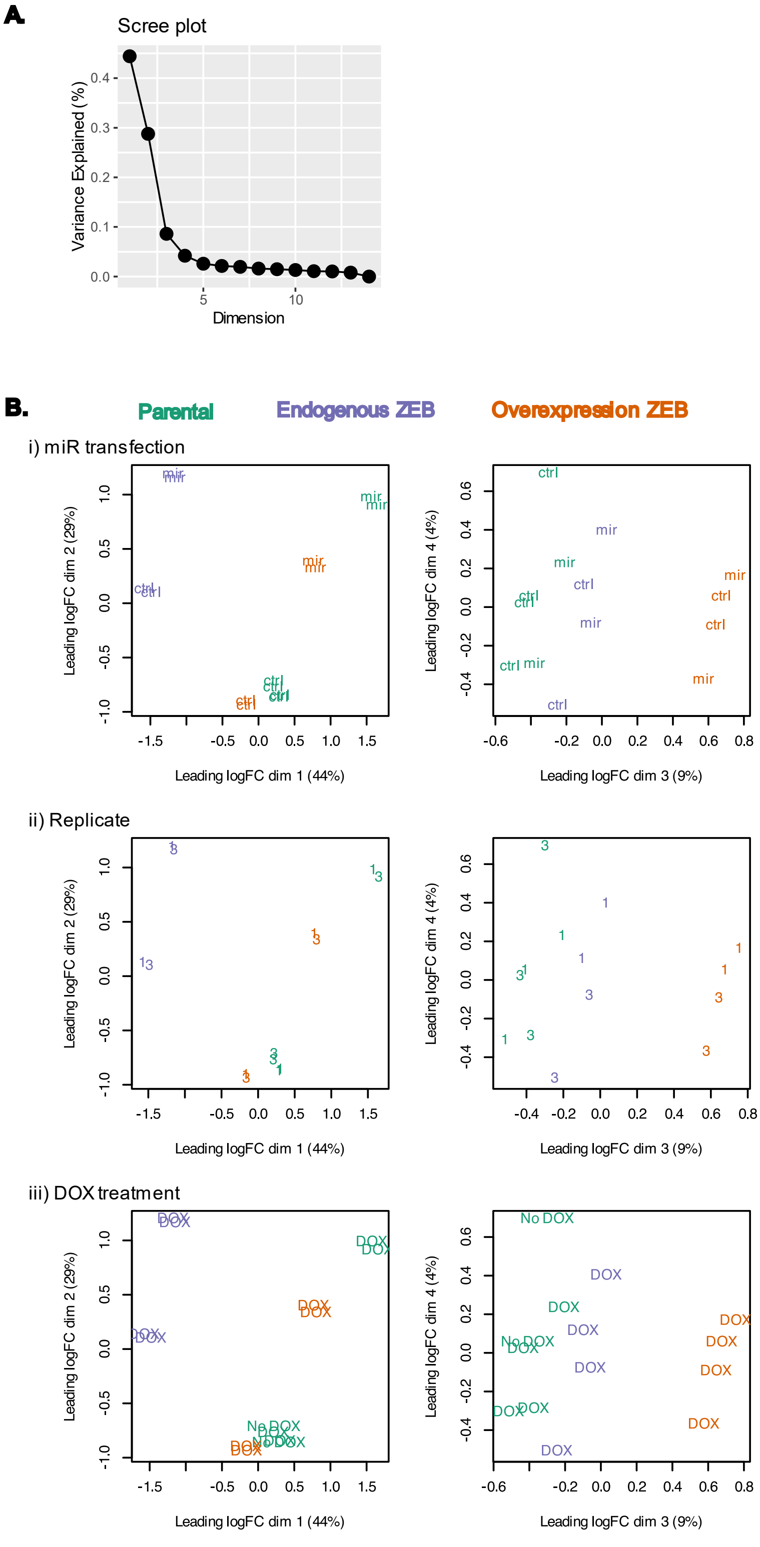

Supplementary Figure 1. Multi-Dimensional Scaling Analysis

A) Scree plot. (B) Loading plots for dimensions 1-4. The samples showed tight clustering of biological replicates (ii) and separation of background and miRNA treatment (i) in the first two dimensions (representing cumulatively 73% of the variance). Samples with and without DOX treatment showed no separation in these dimensions (iii). Dimensions 3 and 4 showed further separation by background but not replicate. Dimension 6 showed separation by biological replicate (data not shown).

A. **Single variable design**

	scrxP	mirxP	scrxOEP	mirxOEP	scrxOEC	mirxOEC	doxNODOX	rep3
Clone2_DOX_200c_1	0	0	0	0	0	1	0	0
Clone2_DOX_200c_3	0	0	0	0	0	1	0	1
Clone2_DOX_Scr_1	0	0	0	0	1	0	0	0
Clone2_DOX_Scr_3	0	0	0	0	1	0	0	1
Parental_DOX_200c_1	0	1	0	0	0	0	0	0
Parental_DOX_200c_3	0	1	0	0	0	0	0	1
Parental_DOX_Scr_1	1	0	0	0	0	0	0	0
Parental_DOX_Scr_3	1	0	0	0	0	0	0	1
Parental_NO-DOX_Scr_1	1	0	0	0	0	0	1	0
Parental_NO-DOX_Scr_3	1	0	0	0	0	0	1	1
Pool_DOX_200c_1	0	0	0	1	0	0	0	0
Pool_DOX_200c_3	0	0	0	1	0	0	0	1
Pool_DOX_Scr_1	0	0	1	0	0	0	0	0
Pool_DOX_Scr_3	0	0	1	0	0	0	0	1

Interaction design - Two variables and interaction terms

	bgP	bgOEP	bgOEC	mir_typemir	doxNODOX	rep3	bgOEP:mir_typemir	bgOEC:mir_typemir
Clone2_DOX_200c_1	0	0	1	1	0	0	0	1
Clone2_DOX_200c_3	0	0	1	1	0	1	0	1
Clone2_DOX_Scr_1	0	0	1	0	0	0	0	0
Clone2_DOX_Scr_3	0	0	1	0	0	1	0	0
Parental_DOX_200c_1	1	0	0	1	0	0	0	0
Parental_DOX_200c_3	1	0	0	1	0	1	0	0
Parental_DOX_Scr_1	1	0	0	0	0	0	0	0
Parental_DOX_Scr_3	1	0	0	0	0	1	0	0
Parental_NO-DOX_Scr_1	1	0	0	0	1	0	0	0
Parental_NO-DOX_Scr_3	1	0	0	0	1	1	0	0
Pool_DOX_200c_1	0	1	0	1	0	0	1	0
Pool_DOX_200c_3	0	1	0	1	0	1	1	0
Pool_DOX_Scr_1	0	1	0	0	0	0	0	0
Pool_DOX_Scr_3	0	1	0	0	0	1	0	0

B.

CDH1:
E(y) = -0.5x1 + 6.2x2 + -1.6x3 + 3.4x4 + -3.1x5 + 0.8x6 + -0.2x7 + -0.1x8

CFL2:
E(y) = 7.4x1 + 5.7x2 + 7.6x3 + 5.8x4 + 7.4x5 + 5.6x6 + 0.2x7 + -0.1x8

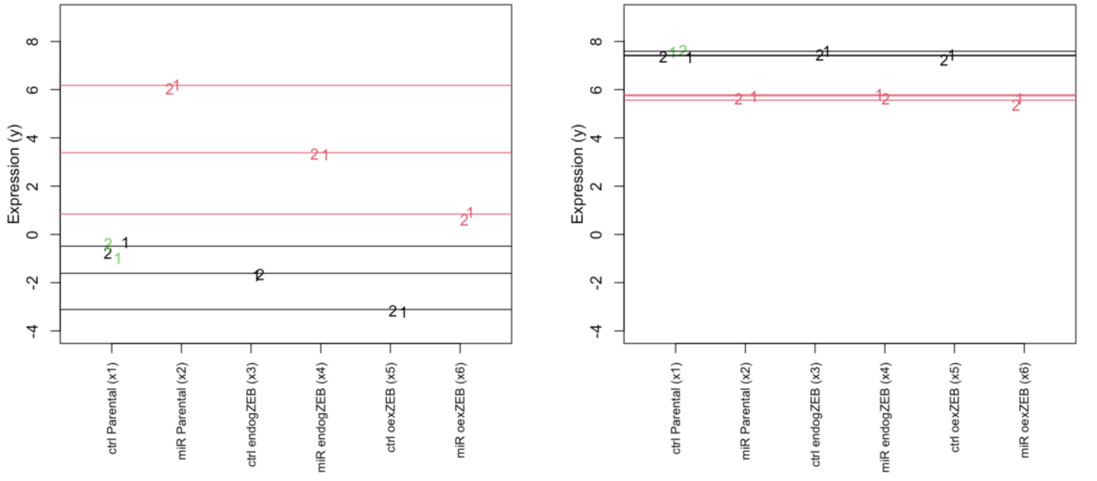

Supplementary Figure 2. Design matricies and fitting linear models to gene expression data

A) The design matrices used for linear models in gene expression analysis highlight the two distinct modelling approaches: (i) single-variable design and (ii) interaction design. Single-variable design: Each column represents a categorical variable (e.g., background type, miRNA type, and treatment conditions). Binary indicators denote group membership for each sample. This design separately quantifies the expression levels present in each sample group. Interaction design: This design incorporates two variables (background type and miRNA type) and their interaction terms. Interaction columns capture the combined effect of background type and miRNA type on gene expression. This approach directly quantifies the varying impacts of the miR in the different ZEB background lines. Both matrices include terms for experimental replicates (e.g., "rep3") and DOX treatment condition (e.g., "doxNODOX"= untreated). B) Fitted coefficient against log2CPM plots: The fit of the models was examined for CDH1 and CFL2 by plotting the first 6 fitted coefficients (horizontal lines) against the CPM values (on the x-axis, with each point shown as a number corresponding to it's biological replicate). The model is structured such that the fitted coefficients describe replicate 1 with dox treatment for each background and miR type. Coefficients and samples are coloured as follows: Red for miR-200, black for control miR, green for samples with no dox treatment. At the top of each plot is shown the fitted equation for the eight coefficients (corresponding to the coefficients in the design matrix). Coefficients x1 to x6 are plotted across the x axis, coefficient x7 corresponds to the effect of "no dox" treatment and coefficient x8 corresponds to the impact of replicate 2

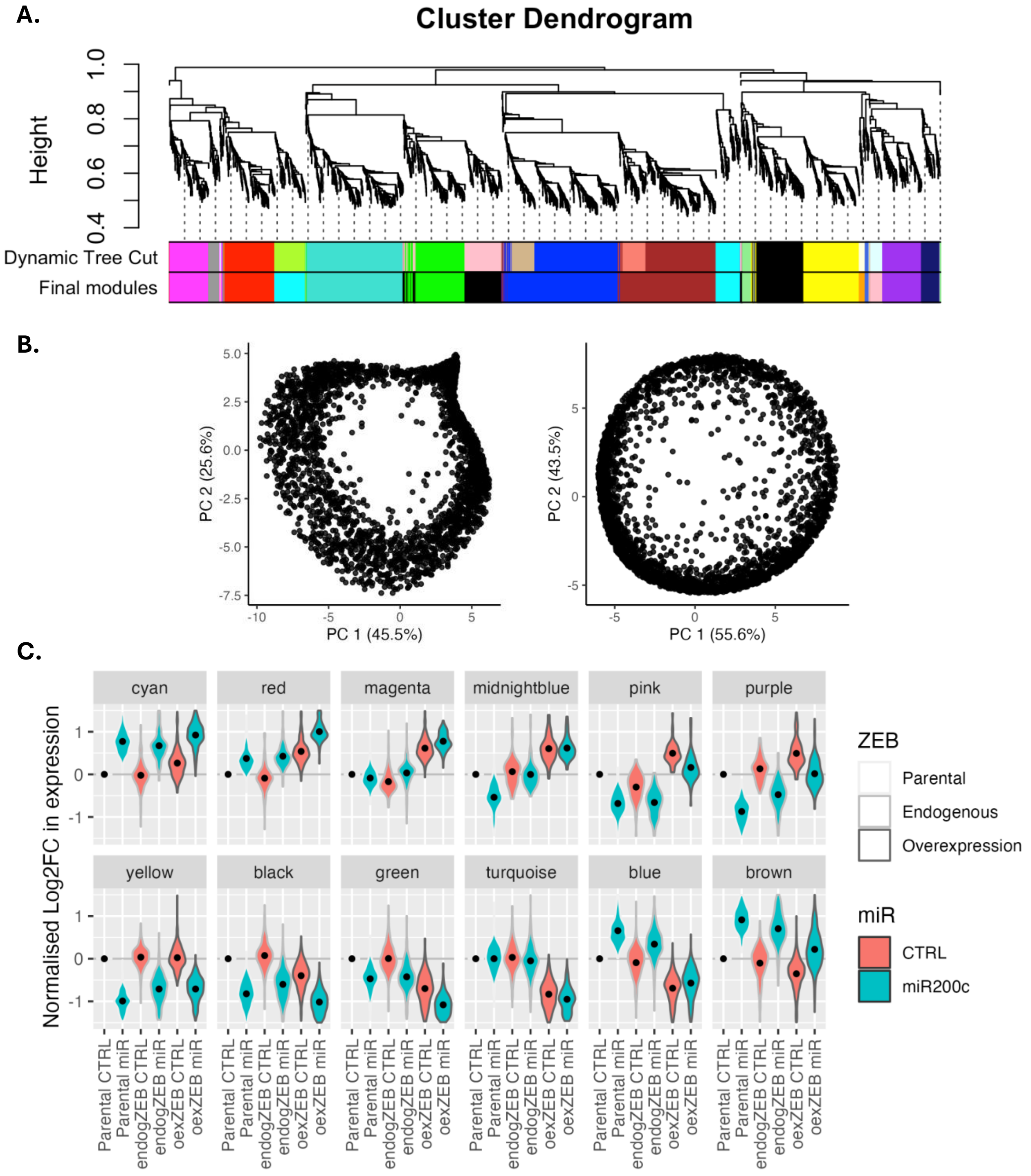

Supplementary Figure 3. Clustering dendrogram and exploratory analyses from Gene Correlation Network Analysis

A) The original modules were examined, similar modules were merged. Following this step, four modules with fewer than 50 genes were discarded: orange, grey60, royalblue and lightgreen. B) PCA analysis of the gene correlation network shows that the circular structure (left) is not caused by soft power data transformation. Instead, performing the analysis with no soft power transformation produces an even more classically circular shape (right), comprising >99% of the total variance. C) Violin plot of relative expression levels of genes in each gene correlation network module. Expression levels are expressed relative to expression in Parental CTRL samples.

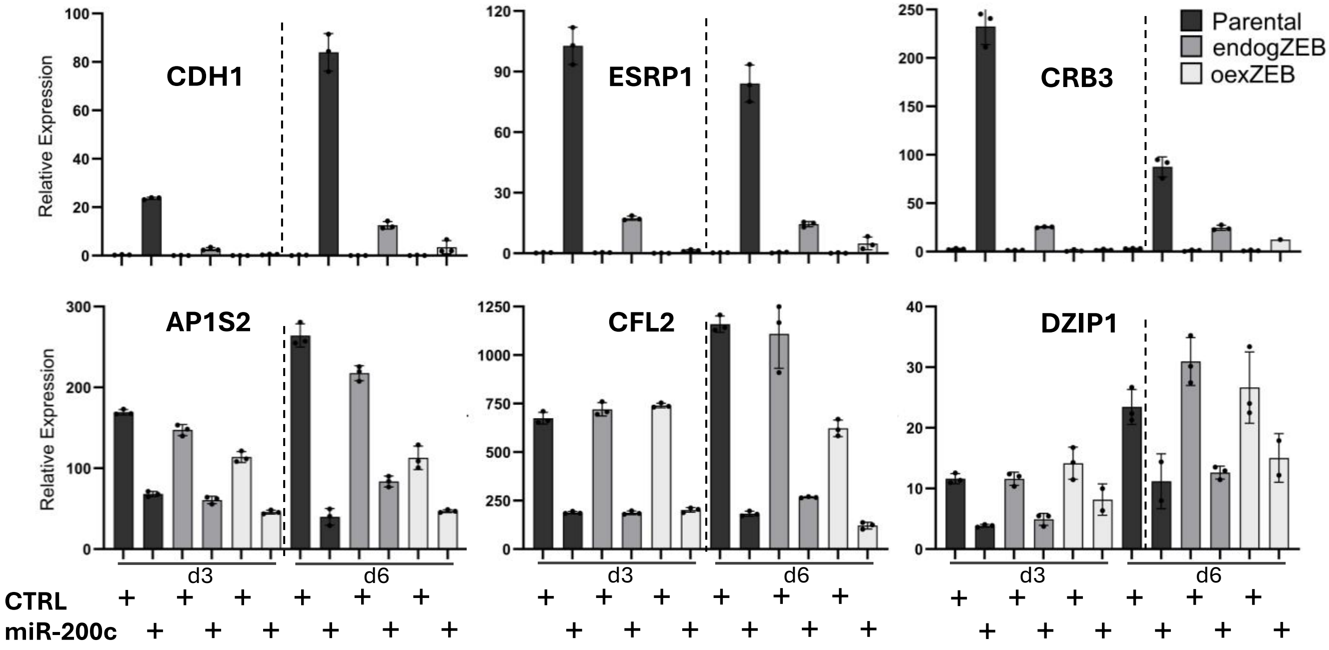

Supplementary Figure 4. miR-200c responsive regulation of epithelial and miRNA target genes

Expression of representative epithelial genes known to be directly repressed by ZEB (CDH1, ESRP1, CRB3) and direct miR-200c-3p target genes (AP1S2, CFL2, DZIP1) in parental, endogZEB and oexZEB MDA-MB-231 cells, either 3 or 6 days post miR-200c-3p transfection.

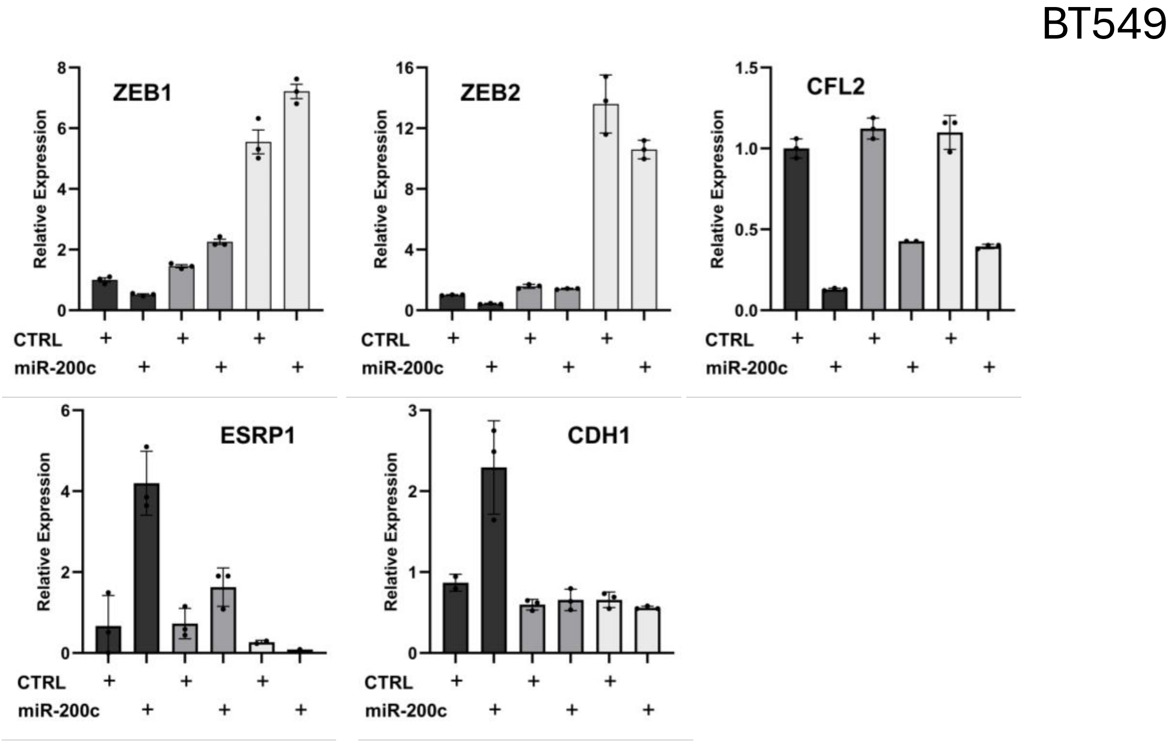

Supplementary Figure 5. miR-200c responsive regulation of epithelial and miRNA target genes in BT549 cells

Expression of representative epithelial genes known to be directly repressed by ZEB (ESRP1, CDH1) and a direct miR-200c-3p target gene (CFL2,) in parental, endogZEB and oexZEB BT549 cells 3 days post miR-200c-3p transfection. All cells were treated with Dox 1 day prior to miRNA transfection. Epithelial genes shown in Supplementary Figure 4 but not represented here were not expressed at sufficient levels in BT549 cells for reliable quantitation.

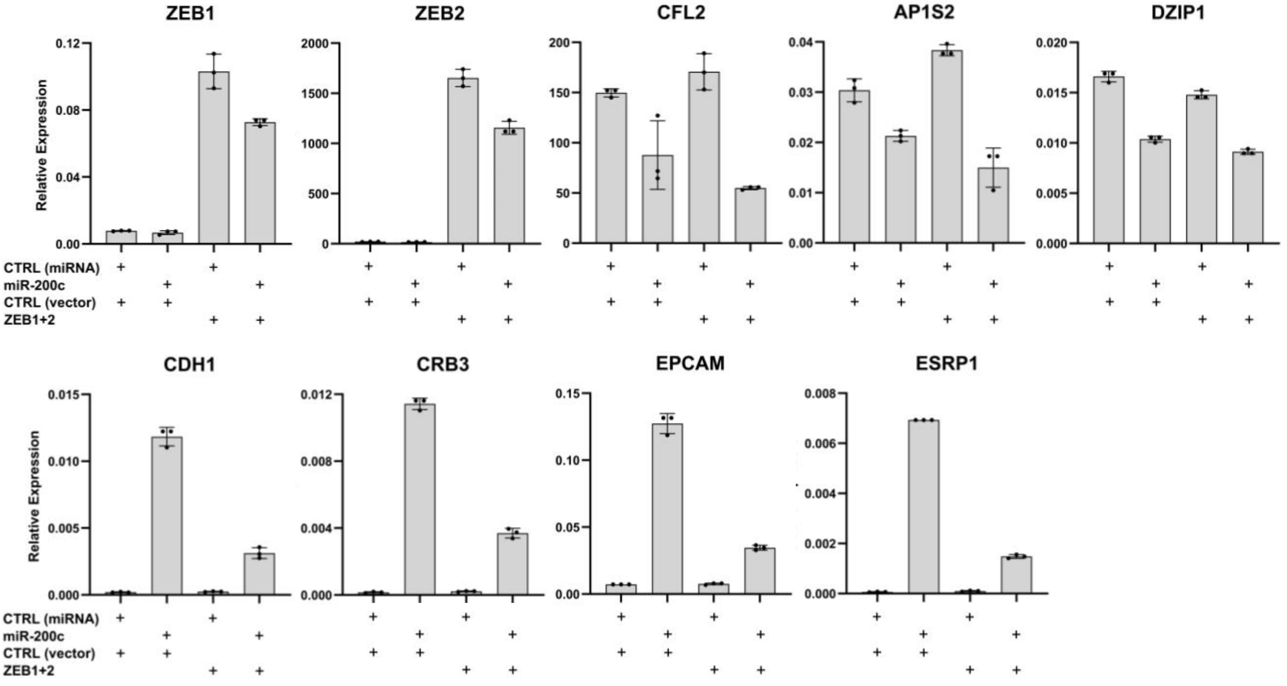

Supplementary Figure 6. miR-200c responsive regulation of epithelial and miRNA target genes in mesHMLE cells

HMLE cells were made to undergo EMT through treatment with TGFβ for 12 days to generate mesHMLE cells. Cells were then transiently transfected with ZEB1 and ZEB2 expression vectors, followed by miR-200c 1 day later. After 3 days, the expression of ZEB1 and ZEB2, direct miR-200c target genes (CFL2, AP1S2, DZIP1) and epithelial markers (CDH1, CRB3, EPCAM and ESRP1) were determined.

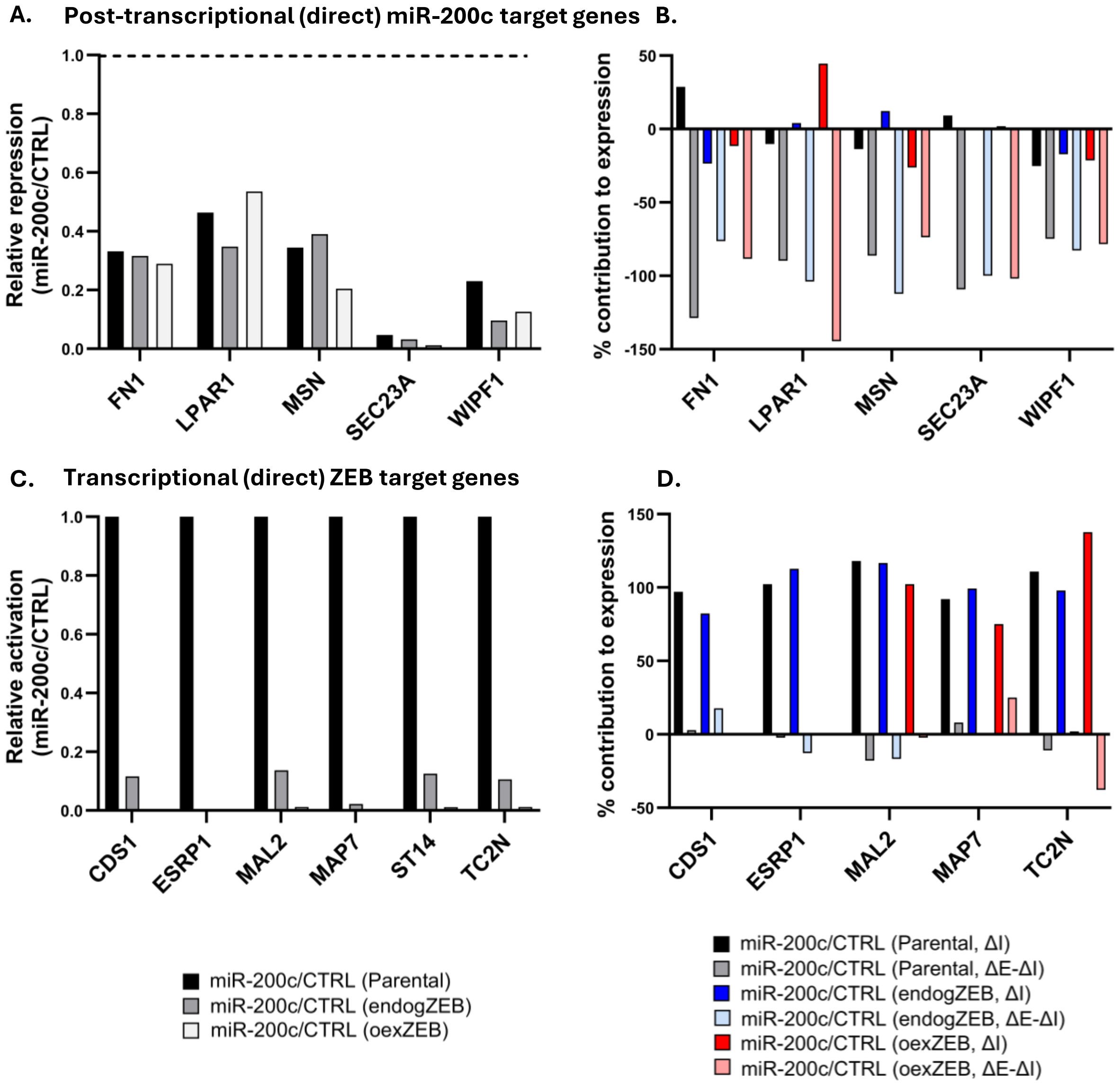

Supplementary Figure 7. miR-200c-3p and ZEB target genes are primarily regulated at post-transcriptional and transcriptional levels respectively

The effectiveness of EISA in this dataset was established by examining the transcriptional (ΔI) or post-transcriptional (ΔE-ΔI) responses of established direct target genes of either miR-200c-3p or ZEB (+/- miR-200c-3p; +/- Dox in parental, endogZEB and oexZEB cell lines). Relative repression (A) or activation (C) of the gene as determined by bulk RNA-seq is shown. B,D) The % contribution of post-transcriptional or transcriptional gene regulation is indicated. Missing bars in (D) indicate insufficient intron-mapping depth for EISA.

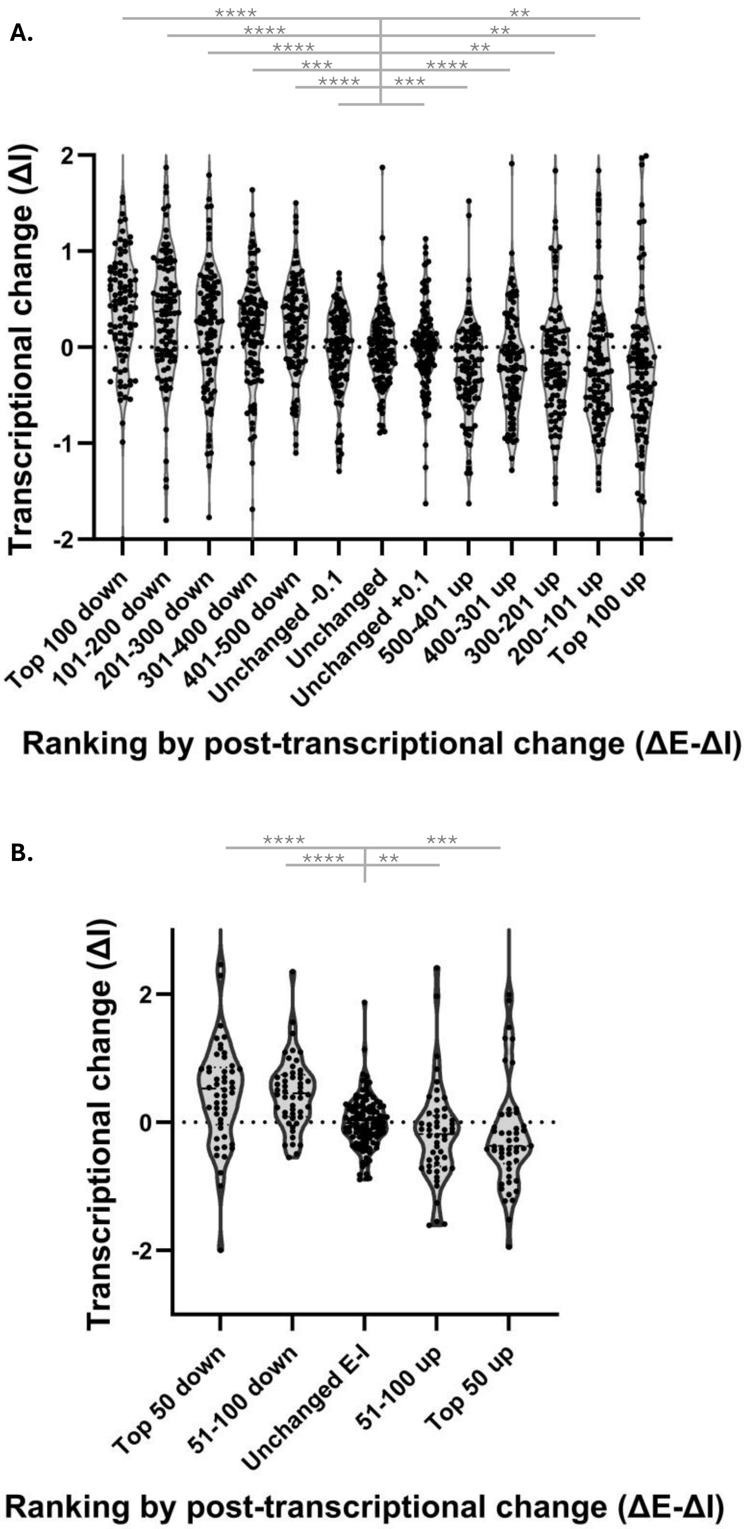

Supplementary Figure 8. Transcriptional and post-transcriptional gene regulatory arms are frequently opposed and act to stablise / buffer gene expression

Relative transcriptional change (ΔI, y-axis) for groups of genes (blocks of 100 (A) or 50 (B) genes) by their degree of post-transcriptional change (ΔE-ΔI) in response to miR-200c-3p. Statistical significance is determined by Kolmogorov-Smirnov test; **** $p<0.0001$; *** $p<0.001$; ** $p<0.01$

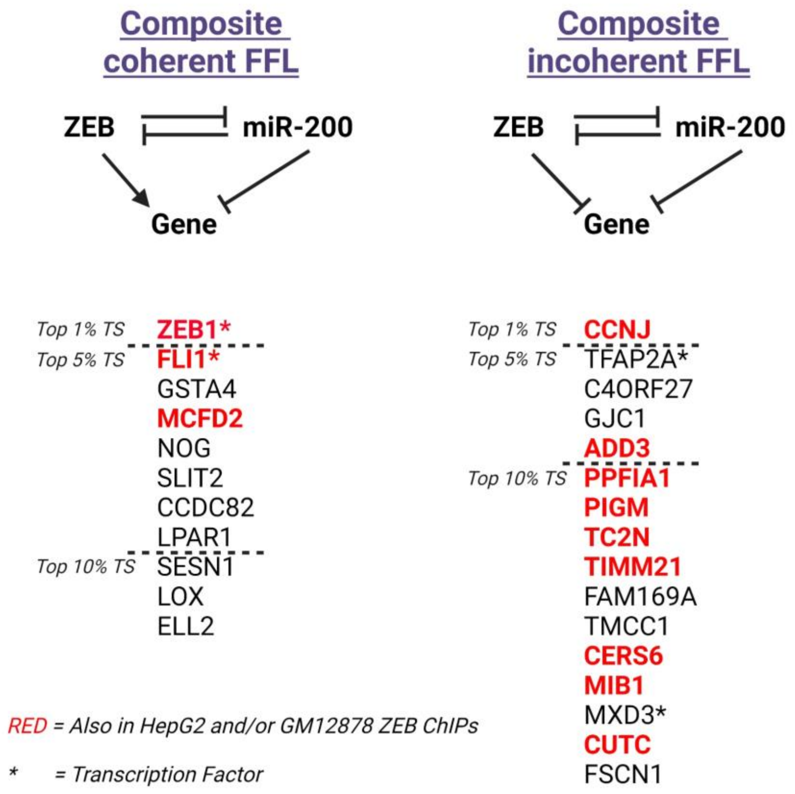

Supplementary Figure 9. Putative coherent and incoherent feedforward loops associated with the miR-200:ZEB axis

For the putative coherent feedforward loops shown, the gene is inhibited and activated by miR-200c-3p and ZEB respectively through their dual direct actions on the gene and on each-other. Such genes would be expected to be mesenchymal in their pattern of expression. For the incoherent feedforward loops shown, miR-200c-3p and ZEB have different effects depending upon whether they are acting directly on the target, or indirectly on each-other. Such regulation is more consistent with buffering or hybrid EMT states. Genes that may participate in such regulatory modules are identified on the basis of the strength of their miR-200c-3p targeting (top 1%,5% or 10% of miR-200c-3p target predictions according to TargetScan) and the presence of ZEB1-bound sites in their promoters according to ZEB1 ChIP-seq in MDA-MB-231 cells, coupled with their activation or repression in the same cell line in response to shRNA-mediated ZEB1 knockdown. For several genes, ZEB1 targeting was further confirmed using independent data derived from HepG2 and/or GM12878 cell ChIP-seq (shown in red). * indicates known transcription factors.

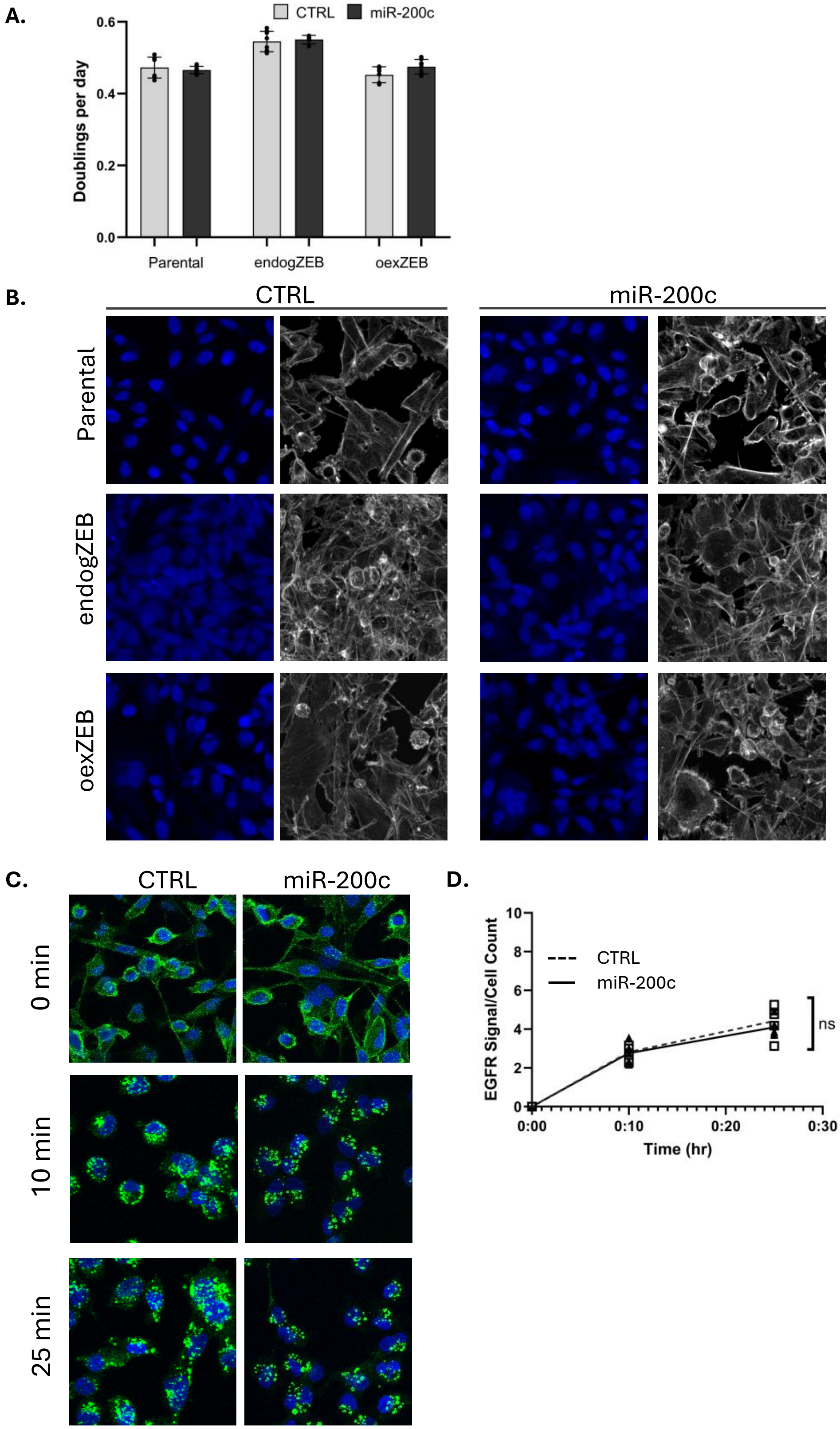

Supplementary Figure 10. miR-200c does not affect proliferation, morphology or EGFR internalisation in MDA-MB-231 cells

A) Proliferation, B) Morphology as assessed by F-actin localisation and C,D) EGFR localisation prior to and after 10 and 25 minutes serum exposure in MDA-MB-231 cells, co-transfected with control RNA or miR-200c-3p.

Full western blots (Fig 3c,4c)

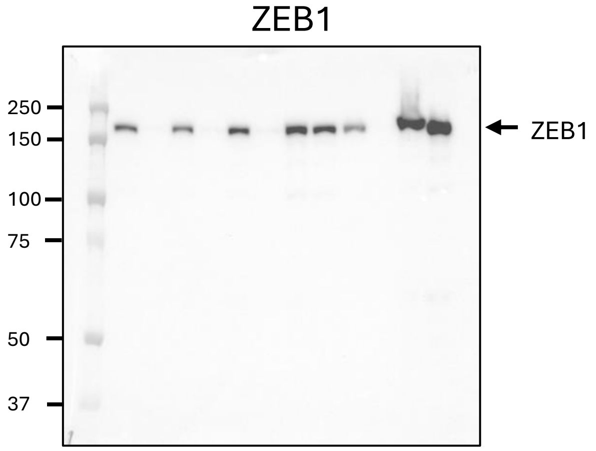

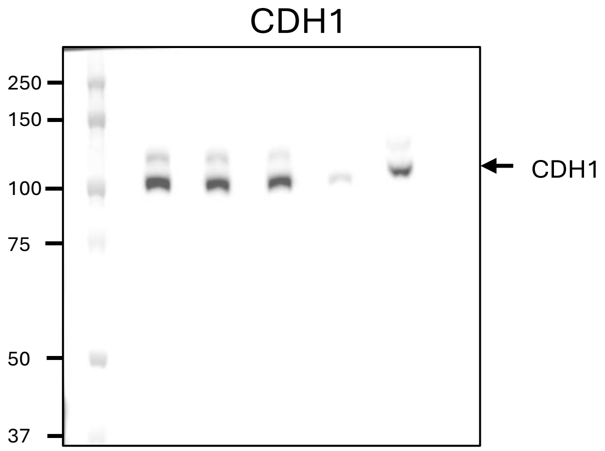

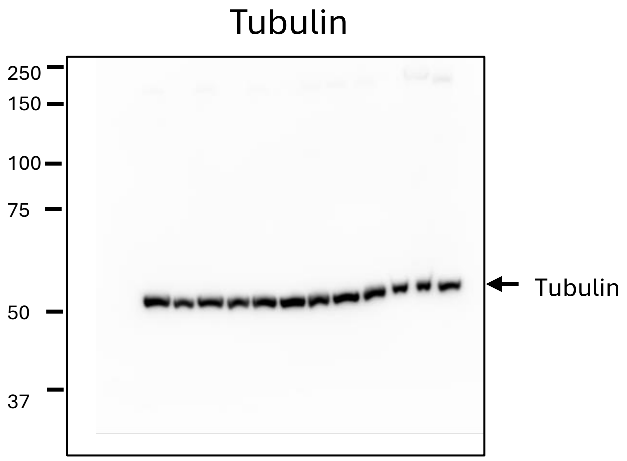

Supplementary Table 1

Application	Target	Sequence	Manufacturer
qPCR	GAPDH	F: CAGTGAGCTTCCCGTTCAG<br>R: ACCCAGAAGACTGTGGATGG	IDT
qPCR	B-Actin	F: CCTGGCACCCAGCACAA<br>R: CTTGCGCTCAGGAGGAGC	IDT
qPCR	ZEB1	F: TTCAAACCCATAGTGGTTGCT<br>R: TGGGAGATACCAAACCAACTG	IDT
qPCR	ZEB2	F: CAAGAGGCGCAAACAAGCC<br>R: GGTTGGCAATACCGTCATCC	IDT
qPCR	CDH1	F: CCCACCACGTACAAGGGTC<br>R: CTGGGGTATTGGGGGCATC	IDT
qPCR	ESRP1	F: AGTCTGCGGACAGAGCATTT<br>R: GGGGATAAGCCATTTCGATT	IDT
qPCR	CRB3	F: CACTGTTTTGCCTTCATCCA<br>R: CGTCTCCTTGGAGTCCTGAG	IDT
qPCR	AP1S2	F: CCTTGAGTGGCGAGA<br>R: TTTCCTGAACTTCCC	GeneWorks
qPCR	CFL2	F: ATGGCTTCTGGAGTTACAGTGA<br>R: GCATATCGGCAATCATTCAGAGG	IDT
qPCR	DZIP1	F: AGTCTTCAACTATTACGACCCCT<br>R: CGTCCGCGTCACTTTTCAC	IDT

Supplementary Table 1. Primer sequences used for qRT-PCR

Number of genes associated with Figure 1

	TS_all	TS_-0.1	TS_-0.2	TS_-0.3	TS_-0.4	TS_-0.5	TS_-0.6	TS_-0.7	TS_-0.8	TS_-0.9
cor_below_-0.5	77	35	24	17	13	10	9	7	5	2
cor_below_-0.45	161	86	48	31	24	18	16	13	10	7
cor_below_-0.4	345	182	94	53	36	25	23	18	13	10
cor_below_-0.35	744	371	201	110	71	41	31	22	17	14
cor_below_-0.3	1643	766	411	241	146	80	54	35	30	23
cor_below_-0.25	3404	1466	740	415	236	123	80	52	45	35
cor_below_-0.2	6438	2684	1295	683	371	196	123	79	61	48
cor_below_-0.15	12042	5011	2329	1185	604	323	186	117	87	67
cor_below_-0.1	22213	9147	4244	2103	1083	566	333	221	150	119
cor_below_-0.05	44490	17843	8154	3869	1946	1013	579	359	242	199
cor_below_0	91358	36093	16279	7401	3568	1810	1011	625	425	325
cor_all	229982	89760	40002	17788	8348	4207	2302	1427	970	723
cor_above_0	138624	53667	23723	10387	4780	2397	1291	802	545	398
cor_above_0.05	87602	33853	14995	6626	3053	1549	823	523	364	256
cor_above_0.1	52426	20268	8974	3993	1859	936	507	321	229	159
cor_above_0.15	29271	11468	5123	2290	1061	534	312	203	137	97
cor_above_0.2	15109	5944	2668	1197	542	256	144	93	59	48
cor_above_0.25	7163	2879	1269	561	270	118	69	42	28	26
cor_above_0.3	3151	1287	533	236	108	50	31	20	13	12
cor_above_0.35	1386	581	225	97	38	15	9	5	5	5
cor_above_0.4	551	236	91	49	21	7	6	3	3	3
cor_above_0.45	239	109	43	23	12	4	4	3	3	3
cor_above_0.5	107	50	22	13	6	3	3	2	2	2

Supplementary Table 2. Numbers of genes associated with Figure 1.

Supplementary Excel file

Supplementary Table 3. MicroRNA : Transcription Factor target prediction and expression correlation

Correlated expression (cor) between microRNAs and targets across the cancer cell line encyclopedia (CCLE). Predicted targeting scores (Targetscan V7.2, TS_score) are shown. Transcription Factors are indicated (TF = TRUE). All putative miRNA : target pairs are shown if they are both in either the top 1% or 5% of target strength prediction and inversely correlated expression.

Supplementary Excel file

Supplementary Table 4. Gene Ontology Analyses

Gene ontology analyses were performed using geneontology.org. Enriched ontologies of each WGCNA module gene and the top 400 differentially expressed genes between HMLE and mesHMLE cells are listed.
